# Nurses’ perception about their role in reducing health inequalities in community contexts

**DOI:** 10.1590/1518-8345.7245.4299

**Published:** 2024-08-30

**Authors:** Jorge Sotelo-Daza, Yaneth Esperanza Jaramillo, Martha Vivas Chacón

**Affiliations:** 1 Universidad del Valle, Facultad de Salud, Cali, Colombia.; 2 Universidad del Área Andina, Facultad de Salud, Armenia, Colombia.; 3 Universidad del Cauca, Facultad Ciencias de la Salud, Popayán, Colombia.

**Keywords:** Nursing, Health Status Disparities, Public Health Nursing, Social Determinants of Health, Health Policy, Qualitative Research

## Abstract

**Objective:**

to understand nurses’ perception about their role in reducing health inequalities in community contexts.

**Method:**

a qualitative study with a phenomenological approach addressed from Heidegger’s Interpretive Theory and the health inequality settings. A total of 18 in-depth interviews were conducted with nurses working in health institutions devoted to community interventions. The following five-phase process was adopted for the qualitative data analysis: Accumulation; Disassembling; Reassembling; Interpretation; and Conclusion.

**Results:**

four main topics that nurses mobilize to mitigate health inequalities in community settings were identified, namely: Competences to create, operationalize, monitor and assess health policies; Leadership and management for health and care processes at the individual and collective levels; Professionals that devise care strategies; and Care approach based on characteristics of the territory.

**Conclusion:**

nurses perceive that their role contributes significantly to reducing health inequalities in community settings based on the creation, operationalization, monitoring and assessment of health policies. From the sociocultural, economic and political characteristics of the territory, they prioritize actions in disadvantaged human groups in order to improve access, opportunities, continuity and quality in health.

## Highlights

**(1)** Nurses’ contributions to reducing health inequalities are significant.

**(2)** Nursing is rooted in equality principles regarding health and social justice.

**(3)** Community health proves to be effective in reducing health inequalities.

**(4)** Communities are essential in the fight against adverse social determinants.

**(5)** Nurses mobilize the elaboration, implementation and assessment of health policies.

## Introduction

Health inequalities are preventable potential differences in terms of health or risks to health, in which health policies can exert an influence among groups of people that can be more or less favored: these differences systematically place disfavored social groups in a more disadvantage position regarding health^1^. Health inequalities are the result of the Social Determinants of Health (SDHs); in other words, of the “circumstances in which people are born, mature, work, live and age, including the broader set of strengths and systems that exert an influence on everyday life conditions”^2^. These inequalities emerge from the social stratification based on richness, power and prestige, whose imbalances produce unfair health results in communities marked by low socioeconomic levels over time^3^. This concept implies economic, political, sociocultural and environmental dimensions; therefore, it has been described that health inequalities can only be eradicated by implementing specific actions on the SDHs^4^. The fight against health inequalities alludes to a challenging Public Health problem, as it determines the well-being conditions of population groups, both in poor and in rich countries^5^.

In this sense, throughout history Nursing has emerged as a profession rooted in the equality principles regarding health and social justice, with the preponderant role of improving people’s, families’ and communities’ health by addressing underlying social and health inequalities^6^ ^)-(^ ^7^; however, acknowledgment of this work by the actors of health systems and society has been set to the background and is affected by discourses and practices from assistance logics that attenuate nurses’ significant contributions in dynamyzing strategies outside hospital settings. 

Focusing interventions based on community health has proved to be an effective strategy to address health inequalities^8^. Local communities play an essential role in the fight against adverse SDHs; in this sense, it is considered crucial to empower them and endow them of knowledge, in addition to attaining the commitment to effectively address health and disease challenges. Community care presents itself as a fundamental Nursing pillar^9^. Through the relationships they establish with community members, nurses can exert a positive influence on health habits and foster the adoption of health protecting behaviors^6^. Likewise, they can empower communities by working on the intersection between its members’ health policies and offer effective and sustainable solutions^10^.

Community Nursing personnel works from systemic perspectives and integrates conceptual and practical elements to provide care at the individual and collective levels^11^. As an essential profession in health promotion and education, Nursing plays a crucial role as a strategic agent to enhance health and minimize risk factors. This achievement is made a reality by promoting healthy everyday life behaviors, in order to contribute to reducing health inequalities^10^ ^),(^ ^12^. Grounded on the human rights and equality principles, Nursing ethics strongly supports these priority functions^6^.

The purpose of this research is to unravel the importance of nurses’ role in reducing health inequalities^13^, supported on scientific evidence. This is grounded on various categories, such as ensuring access to health systems where nurses are the first contact^10^, promoting education and health^14^, providing people-centered care^15^, engaging in community work^16^ and coordinating the care process in a comprehensive way^11^. In this context, the objective of the current research was to understand nurses’ perception about their role in reducing health inequalities in community contexts. 

## Method

### Type of study

This was a qualitative study with a descriptive and exploratory design that resorted to Heidegger’s Interpretive phenomenological approach^17^, which prioritizes nurses’ life experiences and phenomenological perceptions^18^ in relation to performing their role in community settings from a perspective of health inequalities^19^.

### Study *locus* and participants

Purposive or intentional theoretical sampling^20^ was employed in the process to select the participants, which included 18 nurses from 14 Colombian regions working as professionals in community settings. The number of participants was defined with the aim of achieving the point where data collection did not provide any new concepts, categories or relationships. The researchers performed a reflexive and critical analytical process to evaluate the data, in order to establish the theoretical saturation level and density of the information. These criteria were employed to link new participants after a thorough systematic analysis of the data collected. The study period was between March and October 2023.

The following inclusion criteria were considered: working in a community setting and having at least two years of experience in services of the Colombian health system, such as hospitals, municipal or departmental health secretaries, the national Health Ministry and health insurance companies.

Nurses’ influences on mitigating health inequalities were explored to deepen on the meaning of their experiences, as they undergo them in their everyday routines. The study of health inequalities was addressed from a qualitative perspective^19^ and three scopes were considered for the analysis: health; socioeconomic/cultural; and context. The researchers used the Consolidated Criteria for Reporting Qualitative research, COREQ) list^21^.

### Data collection

The data were collected through in-depth interviews that were conducted both in face-to-face meetings at the participants’ workplace and online. All the interviews were audio-recoded and, for those that took place online, in video format via the Teams^®^ platform after obtaining the participants’ consent. Verbatim transcriptions of the recording were made in Word^®^, which allowed performing the pertinent primary analyses. Before initiating data collection, the lead author conducted pilot interviews to ensure clarity, coherence and fluency in writing and sequence of the questions.

The interviews were not subjected to any time restriction and were developed organically, guided by the participants’ commitment to report their life experiences. They lasted between 57 and 93 minutes. The interview script was prepared based on the relevant bibliography. Field notes were taken, both during and after the interviews. No person other than the researchers and the participants was present when the research was carried out. No repeated interviews were conducted. Data collection was considered concluded when sampling sufficiency and saturation were reached.

During the interviews, the participants were asked to provide information such as age, schooling level, occupation, job position and experience time, followed by open questions that were formulated according to an interview guide. All the participants answered an initial question: “Can you tell me your perception about nurses’ role in reducing health inequalities in community contexts?”. Surveys and paraphrases were used during data collection to ensure that the participants provided an adequate view of the questions asked. The following additional questions were also asked: “In your opinion, which aspects of the health system have helped you mitigate health inequalities?”, “What type of resistance have you found in the health system to mitigate health inequalities?”; “Can you describe your experience as a nurse in relation to the care provided to people in social vulnerability situations?”; and “Can you describe how you have addressed these social inequality situations in your job?”. The conversations progressed with comments such as “Continue”, “Please tell me how you experienced it”, “What do you mean by this?”, “Can you think about anything else that helps me understand how it was?” or “Would you like to add anything?”.

One of the components that preserved methodological rigor of the research was related to the research team, which was comprised by nurses from Universidad del Valle, Fundación Universitaria del Área Andina and Universidad del Cauca. They have MSc degrees in Public Health and Epidemiology, and one of them is a PhD in Anthropology. They also make up the faculty in these institutions and have broad experience, both in public health management in services of the health system and in conducting research studies based on qualitative methods. Two of the researchers were women and the third one was a man. The participants were aware of the researchers’ profiles and acknowledged their interest in exploring and understanding nurses’ contributions in different contexts through research projects.

The personal motivations that drove this research emerged during a Nursing symposium, in which the researchers gathered to discuss issues inherent to nurses’ roles in the public health scope. Concerned about nurses’ limited visibility in certain health institutions, the researchers agreed that proposing this research would be a relevant contribution based on their professional objectives and previous experiences in the Nursing practice.

For the participants to take part in the research, the lead author initiated a preliminary contact via email (30 nurses) detailing the study objectives. The individuals that expressed their interest in participating (21 nurses) were contacted via telephone calls to provide them with additional information about the research and coordinating the interview details taking into account their time availability, in addition to scheduling the date and place for the meeting. Eventually, three people decided not to participate in the research due to time constraints.

The participants included in this study worked in institutions belonging to the Colombian health system responsible of managing actions at the community level. These institutions include Departmental (regional) and Municipal (local) Health Secretariats, in addition to public ones in charge of leading public health management processes according to the national health policy through plans, programs and projects. Other participants held leadership positions in low-complexity (Level 1) public hospitals, where health promotion and diseases prevention actions are implemented in rural and urban contexts. In addition, some participants worked in health insurance companies and private bodies responsible of managing risks and leading health assurance in the population.

In the reflexivity framework, the researchers critically acknowledged their role during the entire research process and were aware of their own experiences, values and perspectives to limit their influence in data collection and interpretation. Strategies were adopted to minimize biases from collaboration with other researchers, which allowed receiving feedback, external perspectives and valuable comments. In turn, a continuous critical reflection process was linked, complemented with definition of a data saturation point, use of field diaries and recording of reflections and emotions during the process, as well as adopting a transparent approach in all the research stages.

### Data analysis

In the first stages, the researchers analyzed the interview transcriptions individually^22^ with the purpose of acquiring the overall meaning of the experience. Subsequently, the materials were read a second time to identify the meaning units. The “constant comparative method”^23^ was applied to reach consensus in coding of the categories^24^. The next step was to implement a qualitative data analysis process^25^ in five phases, namely: Accumulation; Disassembling; Reassembling; Interpretation; and Conclusion.

During the Accumulation phase, all 18 transcribed interviews were organized and their integrity was verified. In the Disassembling phase, the data were broken down into smaller elements, which implied a two-way iterative and hermeneutic reading between the parts and the whole. This eased identification of recurrent words, phrases and narratives, which enabled the in-depth analysis in the Reassembling phase. A number of codes emerged from the interpretive exercise during the reassembling process. In the Interpretation process, the reassembled material was used to create a “new story” extracting various topics from the interviews.

The researchers coded the data and jointly identified meaning units. From the relationships between these meaning units, they were grouped into broader and comprehensive categories, which lead to the creation of a system made up of emerging thematic nuclei or meta-categories. In addition, qualitative vectors were identified to interpret the data by means of a sequential and cross-sectional analysis of the meta-categories based on the aforementioned theoretical framework, which provided empirical support to analyze the information. Any and all discrepancies that emerged during the discussion were addressed and solved^25^. The data were managed in Atlas.ti 8.0. The analysis plan was triangulated among the researchers. Five participants were invited to review the categories identified and which reflected their experience, as confirmability process for the categories and codes.

### Reliability

Data reliability was established based on the criteria proposed by Lincoln and Guba^26^, which included credibility, transferability, reliability and confirmability. The researchers sought credibility through their continuous interaction with the participants during data collection and by verifying the transcriptions and codes extracted with the interviewees to guarantee their accuracy. Confirmability was ensured by means of an independent data analysis performed by two of the researchers, who corroborated the findings without resorting to their own hypotheses in data interpretation or excluding any result that contradicted the participants’ opinions. Regarding reliability, all the interviews and data collected were in charge of the same researchers, whereas assessment and confirmation of the findings were reviewed by an external researcher that was familiar with qualitative research. In order to achieve transferability, sampling with maximum diversity in terms of age, both genders and schooling levels was used, in addition to providing a precise report of the participants’ statement to ensure applicability of the findings to other contexts

### Ethical aspects

The research applied ethical principles as per the Declaration of Helsinki^27^, the CIOMS code and Ezekiel Emanuel’s ethical requirements, being considered as a minimum-risk research study. The participants were handed in and signed an informed consent form in which the study purpose was explained in detail and the doubts that emerged about the confidentiality principles were solved. Codes were used to identify each participant so as to preserve their anonymity during all the research stages. The research project was approved by the Ethics Committee of Universidad del Cauca, according to Minute No. 6.1-1.25/06 dated March 2023.

## Results

### Characteristics of the participants

The research participants were 18 nurses aged between 27 and 48 years old, with a mean of 38.8. On average, the interviewees had 13 years of experience. Their sociodemographic characteristics are presented in Figure 1.

**Figure 1:** Sociodemographic characterization of the participants. Nurses’ perception about their role in reducing health inequalities in community contexts. Colombia, 2023

No.	Schooling level	Age	Gender	Region	Years of experience
1	MSc in Epidemiology	36	Female	Cauca	13
2	MSc in Public Health	38	Female	Nariño	16
3	MSc in Public Health	41	Female	Cundinamarca	14
4	MSc in Epidemiology	38	Female	Cauca	16
5	MSc in Health Management	39	Female	Cauca	15
6	MSc in Public Health	48	Male	Valle	21
7	MSc in Management	27	Female	Amazonas	3
8	Professional Nurse	33	Female	Quibdó	5
9	MSc in Hospital Environments	44	Male	Huila	10
10	MSc in Public Health	48	Female	Risaralda	17
11	MSc in Virtual Learning Environments	36	Male	Caldas	20
12	MSc in Epidemiology	37	Female	Quindío	8
13	Specialist in Heath Audits	32	Female	Tolima	6
14	Professional Nurse	35	Female	Tolima	7
15	Professional Nurse	49	Female	Valle	23
16	Professional Nurse	29	Female	Orinoquía	4
17	Specialist in Epidemiology	46	Female	Atlantic	23
18	Specialist in Heath Quality and Audits	43	Female	Goal	14

The participants interviewed perceived that the role they have been performing in several community settings during their professional practice has allowed them to significantly contribute to reducing people’s, families’ and communities’ health inequalities.

**Figure 2:**
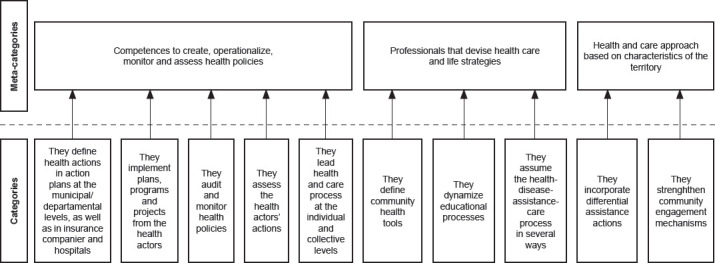
Emerging meta-categories and categories. Nurses’ perception about their role in reducing health inequalities in community contexts. Colombia, 2023

A total of 379 codes emerged from the data analysis. The researchers interpreted the codes independently and the categories were triangulated. Three main topics (meta-categories) were identified: (A) Competences to create, operationalize, monitor and assess health policies; (b) Professionals that devise health care and life strategies; and (c) Health and care approach based on characteristics of the territory. Each meta-category included from two to five categories (Figure 2).

The emerging categories and the health inequality settings in which the nurses identified their role in reducing these inequalities are detailed below.

### Meta-category: Competences to create, operationalize, monitor and assess health policies

In the health policy context, the interviewees perceive that nurses hold job positions that allow them to make decisions in the creation, implementation and assessment of plans, programs and projects related to health care, following the structure of the Colombian health system policies. Nurses’ participation in this scope establishes criteria to define actions that will be included in the mandates set forth in the development plans, both at the municipal and at the departmental levels. This exerts a significant impact on the paths to be followed to allocate health resources.

This meta-category gives rise to five categories, which are described below.

### Category: They define public health actions in action plans at the municipal/departmental levels, as well as in insurance companies and hospitals

Teams led by nurses develop actions that are incorporated into plans, programs and projects to be executed during the Government’s administration (four years), both in the municipal health secretariats and at the departmental level. Likewise, in insurance companies and hospitals, these professionals contribute to defining the strategic processes that will the basis to carry out mission-related initiatives established by the various health system actors in their political efforts. Testimonies like the following ones emerge in this order of ideas:

*In this health system, as nurses we have the possibility of creating the policies that will be implemented in the departments and municipalities. The same happens for first-level hospitals: there we’re thinking in how the administrative and assistance processes are consolidated to advance in ensuring the right to health* […] *I see that, from the EPS (insurance companies), there are a lot of nurses that work every day to guarantee the medicines, consultations and medical exams to their members […] it’s like a network of nurses to achieve people’s well-being from all these health institutions* […] *it’s because of this that I feel I really contribute to reducing those health inequalities.* (ETV1015M-48)

### Category: They implement plans, programs and projects from the health actors

The participants not only perceive their leadership in the action planning phase but also while implementing the operational processes, where they play a crucial role by defining various ways for health actors to perform the actions proposed in the development plans. The implementation of these public policy components is systematic and led by nurses, who, through their role, bring the health system closer to population groups that face greater challenges to access health benefit plans:

*A good number of professionals is mobilized in Nursing; they’re devoted to implementing public health projects in the community; that is really genuine support which ensures that everything is done with quality* […] *because, if you look at other professionals, they lack this technical support, and you may doubt if they’re going to do everything as planned* […] *when you see that there are nurses leading the institutions, you have more confidence, more peace of mind that the intended results for the people will be achieved.* (ETV1002F-27)

### Category: They audit and monitor health policies

From another role, nurses assume the task of assessing the compliance degree of the actions implemented by health system actors. The participants perceived that audits are a crucial element to achieve the system objectives. This implies a continuous and systematic supervision process to ensure that health policies are complied with, in order to guarantee satisfactory results for the population:

*The health secretariats use different follow-up formats and a series of indicators with which we monitor whatever that health institutions have been implementing* […] *this is a very important topic because we make decisions according to these steps forward and work towards improving people’s health* […] *and of course, health equality based on all this evidence.* (ETV1005M-44)

### Category: They assess the health actors’ actions

The participants perceived that the analysis of the information and the progress made in implementing health actions allow them to assess the activities performed by the health actors. This analysis of the compliance degree of the actions leads to adapting the activities according to the population dynamics that characterize the territories, with the objective of identifying improvement opportunities in how the plans are formulated, in order to increase efficiency:

*We’re in charge of providing technical assistance to the municipal health institutions; in this task, we go there and assess their progress in implementing their development plans and monitor everything so that the health programs and budgets allocated are executed as intended* […] *then we perform an assessment to verify if what was projected exerted any effect or not, both with the professionals and with the communities* […] *this face-to-face with people is really exciting and fills you with satisfaction if everything’s gone well* […] *or with anger when you see that things weren’t done with dedication.* (ETV1009M-36)

### Category: They lead health and care processes at the individual and collective levels

According to the health policy guidelines, quality management in health actors is a normative requirement of mandatory compliance. The participants perceive that the nurses lead these policy components in a specific way, based on mobilizing resources, strategies and processes to ensure safety in the provision of health services. They also perceive that ensuring the compliance of care and assistance protocols for people in community environments after a hospitalization or in health promotion and disease prevention actions is an everyday task that contributes to reducing the risks for the population. The implementation of individual health plan in low-complexity hospitals is in charge of nurses, who recognize structures at the community level in order to ease people’s access to health services, focused on health promotion and disease prevention actions. On the other hand, they devise public health plans that include collective interventions and with risk approaches from the departmental and municipal levels. Through their many actions, they implement plans, programs and projects that involve communities in educational processes targeted at disease prevention and adapted to the singularities of each context:

*There’s always some nurse organizing everything so that these processes are complied with; he/she is generally always there watching over the programs so that they keep working, doing follow-up, measuring indicators, providing feedback to the institutions; many times, we even have to watch over the issue of costs and, well, we need to be there as in everything* […] *and you think: this has its advantages, this ensures that things just work.* (ETV1003F-41)

### Meta-category: Professionals that devise health care and life strategies

The participants perceive that nurses devise strategies to implement health and care actions. These professionals have a comprehensive view of the health-disease-assistance-care process; in this sense, they stand out for their strong ontological connection to the specific dynamics of the territory, which they employ to exert a larger impact in reducing health inequalities.

### Category: They define community health tools

The participants report that tools are constantly created to put community management in health into practice. This implies integrating diverse local knowledge, community networks, social engagement and collaboration efforts with institutions from other sectors, such as education, infrastructure and nutrition, among others:

*As a community health nurse, it’s up to you to devise the strategy that best suits the population… mind you, always from the side of the people’s voice and their knowledge... the same happens when joining the other institutions that control the education, nutrition, sports and running water programs, etc.* […] *you do nothing but think: what instrument can I create to improve people’s health?* […] *and the best thing is that people themselves give me hints to make them.* (ETV1012F-35)

### Category: They dynamyze educational processes

One of nurses’ essential roles in the community setting is their contribution to educational processes, where it is noticed that they play a preponderant role as agents that mobilize information and education in order to promote health and prevent diseases by incorporating theories and pedagogical models to improve the teaching/learning of this discipline:

*Basically from the education side, I think that this is where our strength lies* […] *educating in the municipalities and departments, educating the community in general but also being able to guide the professionals that work both in the hospitals and in the municipalities so that, in turn, they can address the themes with their communities.* (ETV1014F-38)

### Category: They assume the health-disease-assistance-care process in several ways

The nurses’ perception about the health-disease-assistance-care process has implications on the way in which actions are addressed in the community environment. This is not only assumed from the health policy perspective but also incorporates the singularities of the territory as an essential element to plan activities adapted to the sociocultural dynamics of each population group:

*I feel that, fortunately, as nurses we consider that health is a complex and broad topic, not only from the clinical perspective, we rather see it as the result of what is mobilized in the entire community* […] *it depends on the social determinants* […] *this view is very important so that, as leaders of the health sector, we advance in strengthening health interventions, especially from community health* […] *and making a reference to what people say and have in their communities.* (ETV1018F-46)

### Meta-category: Health and care approach based on characteristics of the territory

According to the participants’ perspective, nurses take into account the sociocultural, political and economic dynamics of the territories to develop actions in a contextualized way, in line with the parameters established by the health system. This task is fundamental, as it not only implies activism in implement the institutional development plans but also integrates the populations’ realities from various perspectives. This not only includes budgetary and strategic aspects but also local resources that contribute to managing the health process.

### Category: They incorporate differential assistance actions

In the process of providing care to the population, nurses define criteria and designs process that ease the implementation of differentiated assistance actions. These actions are carried out both in the provision of individual services and in the development of collective activities in communities:

*We need to understand these other forms of the health-disease process. For example, the indigenous and afro disharmonies and imbalances, and being able to take part in these other health care constructions* […] *having alternatives or other ways to provide health care is also useful for us in this process of reducing inequalities* […] *when I face these contexts, being in an environment that’s so rich from the cultural point of view, the territory become a talking subject.* (ETV1007F-36)

### Category: They strengthen community engagement mechanisms

Nurses strengthen the community engagement process by establishing relationships with various leaders, organizations and social actors in the territories. These interactions are based on a collaborative dialog that allows agreeing upon the actions approved by consensus with the community. It is in these contexts that health system, population, social determinants and community leadership are integrated to promote the mobilization of actions related to health care:

*Who’s better than us to mobilize the community?* […] *we’re the ones closest to people, the ones that were instilled the value of active listening to coordinate and carry out health activities with people based on their emotions… we organize the strategies with the community according to its needs and, well, we move them forward* […] *I feel that this mobilizes us and makes us feel that we contribute to eliminating inequalities.* (ETV1011F-37)

The participants added the perception that, despite the efforts made by health actors to address health inequalities, substantial challenges persist which hinder systematic progress towards that end. These challenges are related to limited investment in health promotion programs, low wages, discontinuation in the hiring of professionals, barriers in access to health services, excessive bureaucracy in the provision of services, denial of home visits, low availability of services in rural areas and corrupted practices.

On the other hand, although most of the participants had graduate studies, this characteristic did not prove to be an indispensable requirement to perform as nurses that contribute to reducing inequalities in terms of health. In fact, health institutions do not usually ask for specific graduate training requirements in their hiring processes.

## Discussion

Based on the task of understanding the nurses’ perception about their role in reducing health inequalities in community environments, these professionals recognize their contribution to this purpose in the different settings where they carry out their professional practice. This is done through various practices in individual and collective care and in the home and community environments, following a logic that is centered on health promotion and disease prevention. The contributions are made evident in the implementation of health policies that are defined in the development plans of the health actors at the municipal, departmental and national levels.

Despite this reality, the way in which the health system recognizes the nurses’ work in community settings is not fully in line with their perception. In fact, some health systems restrict the interventions targeted at these contexts in strategic, financial human resources and infrastructure terms, prioritizing hospital-centered care processes. This situation discourages the systematic implementation of community health, which leads to limitations in access to health systems, with the corresponding implications in the reduction of inequalities^10^ ^,^ ^12^. Likewise, it exerts a negative repercussion on nurses’ performance when assuming their role, as it affects aspects as their remuneration, job stability, hiring modalities and consistency in the implementation of community health strategies^6^.

In turn, other studies^28^ are consistent with the findings and highlight nurses’ role in reducing health inequalities by addressing the critical health-related challenges at a macro level that persistently affect groups of disadvantaged people based on comprehensively linking prevention, treatment and care processes, with emphasis on people in their life context and on the conditions in which they are born, live, work and age.

Three key scopes are incorporated to elucidate nurses’ perception about their role in reducing inequalities, namely: health, socioeconomic/cultural, and context^19^.

### Health system scope

In addition to evaluation the population’ needs and taking part in the development of health policies, nurses play an essential role in guaranteeing the right to health^16^ ^),(^ ^29^. This implies assuming administrative and management responsibilities targeted at promoting access, use of services, continuity, opportunity and quality in the care provided; principles that permeate any health system. Although this category emerges with strength in the testimonies, studies indicate that nurses are oftentimes excluded from decision-making in the formulation of public policies^30^ ^)-(^ ^31^, which implies limitations in the adoption of a holistic approach towards the health-diseases process in the governance actions, with effects restricted to implementing strategies that promote well-being in people living in unfavorable socioeconomic conditions^6^. Despite these discrepancies, certain studies support the idea that nurses play a fundamental role in the implementation of public health policies when they participate in settings that demand broader decision making^10^.

Nurses stand out for their skills to propose and develop various health care strategies, both at the individual/family level and in communities. From a broad perspective of the health-disease-assistance process, they have competences to analyze social determinants of health, which allows them to act through practices aimed at reducing inequalities according to the specific challenges found in each territory^13^. Nevertheless, addressing the social determinants implies that other sectors assume roles and responsibilities and that nurses’ leading role might be impaired, especially if there is no articulated political practice that defines the parameters through which the health sector actions will proceed along with those by other institutions that contribute to the well-being of population groups^10^.

Monitoring and assessment are essential elements in health systems; they are devoted to evaluating the progress of health activities. Based on data collected from the health sector dynamics, nurses analyze indicators systematically and perform critical assessments of the interventions in terms of their effectiveness. By identifying deviations in the path to achieving objectives, they manage these restrictions and, through improvement processes, they propose strategies that strengthen meeting the essential purposes of the health system^32^. One of the advantages to achieve this essential function of Nursing is these professionals’ effort to maintain close ties with individuals, families and communities alike, as well as with health institutions and the way in which the health system is mobilized based on territorial logic^6^. A complex aspect inherent to this dynamics refers to the institutions’ ability to meet the objectives established in the development plans. These objectives are somehow conditioned by the financial and structural situations of each actor in the health scope. This generates tensions in the nurses’ monitoring processes while performing follow-ups and evaluations^12^.

A fundamental element of nurses’ role is their ability to lead health management groups, in order to promote both individual and collective actions. This skill is reinforced by the Nursing professionals’ systematic immersion in the territories. This practice allows them to identify the singularities of both the context and the health institutions, which in turns serves them as a basis to promote actions from public health^33^ and advanced nursing practice^34^. Despite the relevance of these practices, group leadership is affected by challenges in coordinating the actions with professionals from other disciplines due to differences in terms of approaches and perspectives to assume the care processes^35^. On the other hand, there are frequent limitations in community engagement due to unawareness, distrust in health services and limited resources to active participation, an aspect that can destabilize the path to achieving health purposes^6^. Limitations in funding of health teams, resistance to organizational changes and high workloads are added to the aforementioned, which can impair nurses’ leadership actions^12^.

It is essential to note nurses’ role as key agents in the promotion of educational processes from different angles^6^. In the community, they play a relevant role in fostering healthy lifestyle and rigorously collaborate with community leaders to gather and mobilize population groups. This practice supports continuity of the educational process, whether from individual actions in health services or through collective actions developed in community environments. Likewise, thy provide technical counseling to other professionals working in the health system in order to dynamyze the health policy, as corroborated in other studies^7^. This role can be affected by environmental barriers related to the limited time to carry out education actions, to discontinuity of the health promotion plans and projects, to the health professionals’ limited pedagogical competences and to the communication-related restriction due to local dialects or cultural barriers that the health system addresses and incorporates in a limited way^36^.

### Socioeconomic/Cultural scope

Nurses mobilize actions that exert an influence on the social environments based on dynamyzing processes centered on public health, with the purpose of reducing the adverse effects caused by diseases and make progress in mitigating health inequalities. This role positions nurses as change agents in organization of society and as actors that implement health goals based on scientific evidence^35^ ^),(^ ^37^, in line with the social reality needs. Likewise, nurses perform thorough analyses to identify and select the most disfavored population groups, to then focus their efforts on improving access to the health system for these groups. This prioritization extends to population segments such as women, older adults, children, immigrants, ethnic groups and people with various sexual orientations, among others. This process highlights the relevance of the contribution made by Nursing to social justice and to advocating for the rights of vulnerable group^6^ ^),(^ ^13^ as a necessary response to health inequalities, in addition to noting the social responsibility that lies on these health professionals. This situation would not be problematic if health systems adequately acknowledged nurses’ role in this complex sociopolitical structure and managed to establish public policy elements that encourage these professionals’ performance. These elements might include wage improvements, definition of leadership roles, graduate training opportunities and health models that strengthen community care^6^.

Nurses manage to adapt health and care actions to the specific characteristics of each territory as an integral part of their work in the community, considering the cultural, social, economic and symbolic assets as resources to drive initiatives from the health sector. Given that, these professionals are immersed in the singularities of the contexts where they work and adopt an in situ approach that recognizes the territory as a dynamic element that exerts an influence on the health, disability and mortality patterns^5^ ^),(^ ^8^. The challenge set forth by this situation refers to the limitations in the number of professionals that choose to work in these environments, especially in rural or peri-urban areas. In those contexts, the sociocultural and political conditions offer few opportunities for developing and strengthening their professional career^7^.

The territory is also recognized as an essential component for health care and life, susceptible to being shaped to achieve optimal health. These care logics in the territory in charge of nurses are not limited to a strictly technical perspective; they incorporate a solid community component^7^ that strengthens the mobilization of processes and leadership in the community. As they acknowledge these dynamics, nurses face the reality that the entire system does not operate following this integrated logic. Rather than that, the actions are oftentimes carried out in a fragmented way, adapting to the contingencies inherent to the epidemiological dynamics of the population groups served. This situation implies and additional effort on the part of Nursing professionals, who must face both the challenges inherent to community work and the coordination of health teams under a collective action premise^6^ ^),(^ ^12^.

### Context scope

The nurses identify the disadvantages faced by individuals, families and communities and make decisions with the purpose of easing the care to be provided, taking into account the life path of each population group. In this process, they focus on the most socially and economically vulnerable groups, considering factors such as the sociohistorical context, the policies applied, schooling levels and the social/political violence dynamics that characterize the territories. As they acknowledge these categories, they make decisions to implement health action with special emphasis on the care provided to population groups with complex social determinants that limit access to health services. These actions are prioritized in territories marked by difficulties regarding geographical access, areas affected by sociopolitical conflicts, low socioeconomic levels and limitations in the availability of public services. Although various studies support these findings^10^ when they state that, in most countries of the world, nurses are change agents that provide holistic support to those whose rights and needs are overlooked due to their social situation and are left invisible before society, this situation generates challenges for the professionals to continue performing their activities in these complex settings, where they will require additional elements to support not only the guarantee of health care access but also continuous actions to encourage nurses to proceed according to this dynamics that challenges their permanence in the territory and, due to health system, fulfillment of the nurses’ role is compromised in complex settings^7^ ^),(^ ^12^ ^),(^ ^38^.

The contribution made by this study broadens the body of scientific evidence related to nurses’ ability to exert an influence on reducing health inequalities, as well as their fundamental role in settings where community health and care are mobilized. These settings highlight nurses’ resilience, although the health policies enforced in the country favor an assistance approach. The study provides grounds for devising and operationalizing policies targeted to strengthening nurses’ performance and emphasize their competences and leadership to address health inequalities and improve access, fairness and quality in health care, especially for socioeconomically and culturally disadvantaged groups.

Among the study limitations, it is important to note that the research is circumscribed to a group of nurses in Colombia and that, consequently, the perceptions and experiences described are restricted to the specific context that was object of analysis. In this sense, the need to conduct studies in other contexts and to expand the sample is set forth, with the purpose of strengthening the body of evidence.

## Conclusion

The nurses perceive that they significantly contribute to reducing health inequalities in community settings through their everyday professional practice in different actors of the health system. These contributions are based on the conception, design, operationalization, monitoring and assessment of actions linked to the plans, programs and projects defined in health policies. Both at the regional and at the local level, these policy elements are led by nurses, who, leaning on their training for managing projects and devising strategies, endow the health system with dynamism.

Nurses’ mobilization of actions in community settings is determined by sociocultural, political and economic characteristics inherent to each population group. These variables are used as criteria to prioritize the groups in situations of vulnerability, with the objective of improving their access to health services and reducing inequalities.
