# Supplementary material for: Nurses’ perception about their role in reducing health inequalities in community contexts
Source: Rev Lat Am Enfermagem. 2024 Aug 30;32:e4299. doi: 10.1590/1518-8345.7245.4299 (PMC11368067; doi:10.1590/1518-8345.7245.4299)
Supplement: Translation

# Translation: Percepção dos enfermeiros sobre o seu papel na redução das desigualdades em saúde em contextos comunitários

**DOI:** 10.1590/1518-8345.7245.4300

**Authors:** Jorge Sotelo-Daza, Yaneth Esperanza Jaramillo, Martha Vivas Chacón

**Affiliations:** 1 Universidad del Valle, Facultad de Salud, Cali, Colômbia.; 2 Universidad del Área Andina, Facultad de Salud, Armênia, Colômbia.; 3 Universidad del Cauca, Facultad Ciencias de la Salud, Popayán, Colômbia.

**Keywords:** Enfermagem, Disparidades nos Níveis de Saúde, Enfermagem em Saúde Pública, Determinantes Sociais da Saúde, Políticas de Saúde Pública, Pesquisa Qualitativa

## Abstract

**Objetivo:**

compreender a percepção dos enfermeiros sobre o seu papel na redução das desigualdades em saúde em contextos comunitários.

**Método:**

estudo qualitativo com abordagem fenomenológica abordado a partir da teoria interpretativista de Heidegger e das áreas das desigualdades em saúde. Foram realizadas 18 entrevistas em profundidade com enfermeiros que trabalham em instituições de saúde de intervenção comunitária. Para análise dos dados qualitativos foi adotado um processo de cinco fases: Acumulação, Desmontagem, Remontagem, Interpretação e Conclusão.

**Resultados:**

foram identificados quatro temas principais que mobilizam os enfermeiros para mitigar as desigualdades em saúde em ambientes comunitários: Competências para construir, operacionalizar, monitorar e avaliar políticas de saúde; Liderança e gestão de processos de saúde e cuidados em nível individual e coletivo; Profissionais que geram estratégias para o cuidado e Abordagem de cuidado baseada nas características do território.

**Conclusão:**

os enfermeiros percebem que o seu papel contribui significativamente para a redução das desigualdades em saúde em ambientes comunitários a partir da concepção, operacionalização, monitoramento e avaliação de políticas de saúde. Com base nas características socioculturais, econômicas e políticas do território, priorizam ações em grupos humanos desfavorecidos, a fim de melhorar o acesso, a oportunidade, a continuidade e a qualidade na saúde.

## Destaques

**(1)** Os enfermeiros contribuem significativamente para a redução das desigualdades em saúde.

**(2)** A enfermagem está arraigada em princípios de equidade em saúde e justiça social.

**(3)** A saúde comunitária marca a eficácia na redução das desigualdades em saúde.

**(4)** As comunidades são essenciais na luta contra os determinantes sociais adversos.

**(5)** Os enfermeiros mobilizam a concepção, o funcionamento e a avaliação das políticas de saúde.

## Introdução

As desigualdades na saúde são diferenças potenciais evitáveis na saúde ou nos riscos para a saúde, em que as políticas sanitárias têm a capacidade de influenciar grupos de pessoas que podem ser mais ou menos favorecidos. Estas diferenças colocam sistematicamente os grupos sociais desfavorecidos em maior desvantagem em termos de saúde^1^. As desigualdades em saúde são o resultado dos Determinantes Sociais da Saúde (DSS), ou seja, das “circunstâncias em que as pessoas nascem, crescem, trabalham, vivem e envelhecem, incluindo o conjunto mais amplo de forças e sistemas que moldam as condições da vida cotidiana”^2^. Estas desigualdades surgem da estratificação social baseada na riqueza, no poder e no prestígio, cujos desequilíbrios produzem resultados de saúde injustos em comunidades de baixo nível socioeconómico ao longo do tempo^3^. Este conceito envolve dimensões económicas, políticas, socioculturais e ambientais, portanto, afirma-se que só é possível eliminar as desigualdades em saúde por meio de ações sobre os DSS^4^. O combate às desigualdades em saúde refere-se a um problema desafiador de saúde pública, pois determina as condições de bem-estar das populações tanto nos países pobres quanto nos ricos^5^.

Nesta noção, ao longo da história a Enfermagem emergiu como uma profissão enraizada nos princípios da equidade na saúde e da justiça social, cujo papel predominante tem sido melhorar a saúde dos indivíduos, famílias e comunidades, abordando as desigualdades sociais e de saúde subjacentes^6^ ^)-(^ ^7^. Contudo, o reconhecimento deste trabalho pelos atores dos sistemas de saúde e da sociedade tem sido relegado e afetado por discursos e práticas de lógica assistencial que atenuam as contribuições relevantes dos enfermeiros na dinamização de estratégias fora dos ambientes hospitalares.

O direcionamento de intervenções baseadas na saúde comunitária tem se revelado uma estratégia eficaz para abordar as desigualdades em saúde^8^. As comunidades locais desempenham um papel essencial no combate aos DDS adversos, neste sentido, é essencial empoderá-las e dotá-las de conhecimentos para assumir o compromisso de enfrentar eficazmente os desafios da saúde e da doença. O cuidado comunitário apresenta-se como pilar fundamental da Enfermagem^9^. Por meio das relações que estabelecem com os membros da comunidade, os enfermeiros podem influenciar positivamente os comportamentos de saúde e incentivar a adoção de comportamentos protetores da saúde^6^. Da mesma forma, podem capacitar as comunidades trabalhando na intersecção das políticas de saúde dos seus membros e fornecendo soluções eficazes e sustentáveis^10^.

A equipe de enfermagem da comunidade atua a partir de perspectivas sistêmicas e integra elementos conceituais e práticos para prestar cuidados em nível individual e coletivo^11^. Sendo uma profissão essencial na promoção e educação em saúde, a Enfermagem desempenha um papel decisivo como agente estratégico para melhorar as condições de saúde e minimizar os fatores de risco. Essa conquista se materializa na promoção de comportamentos saudáveis no cotidiano, a fim de contribuir para a redução das desigualdades em saúde^10^ ^),(^ ^12^. Baseada nos princípios dos direitos humanos e da equidade, a ética da Enfermagem apoia solidamente estas funções prioritárias^6^.

O propósito desta pesquisa reside em desvendar a importância do papel do enfermeiro na redução das desigualdades em saúde^13^, com o aval de evidências científicas. Este objetivo se apoia em diversas categorias: garantir o acesso ao sistema de saúde onde o enfermeiro atua como primeiro contato^10^, promover a educação e a saúde^14^, prestar cuidados centrados nas pessoas^15^, participar de trabalhos comunitários^16^ e coordenar o processo de cuidado de forma integral^11^. Neste contexto, o objetivo desta pesquisa foi compreender a percepção dos enfermeiros sobre o seu papel na redução das desigualdades em saúde em contextos comunitários.

## Método

### Tipo de estudo

Estudo qualitativo, descritivo e exploratório, sendo utilizada a abordagem fenomenológica interpretativista de Heidegger^17^, que prioriza as experiências vividas e as percepções fenomenológicas^18^ dos enfermeiros em relação ao desempenho de seu papel em ambientes comunitários, partindo da perspectiva de desigualdades em saúde^19^.

### Local do estudo e participantes

No processo de seleção dos participantes foi utilizada uma amostragem teórica proposital ou intencional^20^, que incluiu 18 enfermeiros que atuavam como profissionais em contextos comunitários de 14 regiões da Colômbia. O número de participantes foi definido com o objetivo de chegar ao ponto em que a coleta de dados não fornecesse novos conceitos, categorias ou relações. Os pesquisadores realizaram um processo analítico reflexivo e crítico para avaliar os dados, a fim de estabelecer o nível de saturação teórica e densidade das informações. Esses critérios foram utilizados para vincular novos participantes após uma análise sistemática minuciosa dos dados coletados. O período de estudo estendeu-se entre março e outubro de 2023.

Os critérios de inclusão considerados foram os seguintes: trabalhar em contexto comunitário e ter pelo menos dois anos de experiência em serviços do sistema de saúde colombiano como hospitais, secretarias municipais ou departamentais de saúde, Ministério da Saúde e seguradoras de saúde.

A influência dos enfermeiros na mitigação das desigualdades em saúde foi explorada para aprofundar o significado de suas experiências, conforme são vivenciadas em sua atuação cotidiana. O estudo da desigualdade em saúde foi abordado sob uma perspectiva qualitativa^19^ sendo consideradas três áreas para análise: a área da saúde, a área socioeconômica/cultural e o contexto. Os pesquisadores utilizaram o *checklist Consolidated Criteria for Reporting Qualitative Research* (COREQ)^21^.

### Coleta de dados

Os dados foram coletados por meio de entrevistas em profundidade, realizadas tanto em encontros presenciais nos locais de trabalho dos participantes quanto *online*. Todas as entrevistas foram gravadas em áudio e, no caso das entrevistas on-line, em formato de vídeo pela plataforma *Teams* ^*®*^ após obtenção do consentimento dos participantes. As transcrições literais das gravações foram feitas no programa *Word* ^*®*^ , o que permitiu a realização das correspondentes análises primárias. Antes de realizar a coleta de dados, o autor principal realizou entrevistas piloto, a fim de garantir clareza, coerência e fluidez na redação e sequência das questões.

As entrevistas não tiveram restrição de tempo e se desenvolveram de forma orgânica, pautadas pelo comprometimento dos participantes no relato de suas experiências. Sua duração variou entre 57 a 93 minutos. O roteiro de entrevista foi desenvolvido com base na literatura pertinente. Foram realizadas anotações de campo durante e após as entrevistas. No momento da investigação não havia outras pessoas presentes, além dos pesquisadores e participantes. Não foi realizada nenhuma entrevista repetida. A coleta de dados foi encerrada ao ser atingidas a suficiência e saturação amostral.

Durante as entrevistas, os participantes foram solicitados a fornecer informações como idade, escolaridade, ocupação, cargo e tempo de experiência, seguidas de perguntas abertas realizadas de acordo com um roteiro de entrevista. Todos os participantes responderam a uma pergunta inicial: “Você poderia informar como percebe o papel dos enfermeiros na redução das desigualdades em saúde em contextos comunitários?”. Foram utilizadas sondagens e paráfrases durante a coleta de dados para garantir que os participantes fornecessem informações adequadas sobre as perguntas realizadas. Outras questões colocadas foram as seguintes: “Na sua percepção, que aspectos do sistema de saúde tornaram mais fácil a mitigação das desigualdades em saúde?”, “Que tipo de resistência encontrou no sistema de saúde para mitigar as desigualdades em saúde?”; “Você poderia descrever sua experiência como enfermeiro em relação ao cuidado de pessoas em situação de vulnerabilidade social?” e “Você poderia descrever como você abordou essas situações de desigualdade social no seu trabalho?” A conversa progrediu com comentários como “Continue”, “Por favor, conte-me mais sobre como foi para você...”, “O que você quer dizer com esta afirmação?”, “Você consegue pensar em mais alguma coisa que me ajudasse a entender como foi?” ou “Gostaria de adicionar mais alguma coisa?”.

Um dos componentes que garantiu o rigor metodológico da pesquisa relacionou-se à equipe de pesquisadores formada por enfermeiras da *Universidad del Valle*, da *Fundación Universitaria del Área Andina* e da *Universidad del Cauca*. A equipe de pesquisadores possui formação em nível de Mestrado em Saúde Pública e Epidemiologia, sendo um deles Doutor em Antropologia. Os pesquisadores também atuam como professores nessas instituições e possuem ampla experiência tanto na gestão da saúde pública em serviços do sistema de saúde, quanto na realização de pesquisas utilizando métodos qualitativos. Dois dos pesquisadores eram mulheres, e um era homem. Os participantes conheciam o perfil dos investigadores e reconheciam o seu interesse em explorar e compreender as contribuições dos enfermeiros em diferentes contextos por meio de projetos de investigação.

As motivações pessoais que impulsionaram esta pesquisa surgiram durante um simpósio de Enfermagem, no qual pesquisadores se reuniram para discutir questões pertinentes à atuação do enfermeiro no campo da saúde pública. Preocupados pela escassa visibilidade dos enfermeiros em determinadas instituições de saúde, os pesquisadores concordaram em que a proposta desta pesquisa seria uma contribuição relevante com base nos seus objetivos profissionais e experiências anteriores na prática da Enfermagem.

Para que os participantes participassem da pesquisa, a primeira autora iniciou um contato preliminar via e-mail (30 enfermeiros), no qual foram detalhados os objetivos do estudo. As pessoas que manifestaram interesse em participar (21 enfermeiros) foram contatadas por telefone para fornecer informações adicionais sobre a pesquisa e coordenar os detalhes da entrevista, levando em consideração a disponibilidade de tempo, bem como para combinar data e local de encontro. Finalmente, três pessoas decidiram não participar da pesquisa por falta de tempo.

Os participantes deste estudo trabalhavam em serviços do Sistema de Saúde Colombiano responsáveis pela gestão de ações em nível comunitário. Estas entidades incluem Secretarias Departamentais de Saúde (regionais) e Secretarias Municipais de Saúde (locais), bem como instituições públicas encarregadas de liderar os processos de gestão da saúde pública de acordo com a política nacional de saúde por meio de planos, programas e projetos. Outros participantes ocuparam cargos de liderança em hospitais públicos de baixa complexidade (nível 1) onde são implementadas ações de promoção da saúde e prevenção de doenças em ambientes rurais e urbanos. Além disso, alguns participantes trabalharam em seguradoras de saúde e entidades privadas responsáveis pela gestão de riscos e liderança de seguros de saúde da população.

No que tange à reflexividade, os investigadores reconheceram criticamente o seu papel ao longo do processo de pesquisa e estavam conscientes das suas próprias experiências, valores e perspectivas para limitar a sua influência na coleta e interpretação de dados. Foram adotadas estratégias para minimizar o viés a partir da colaboração com outros pesquisadores, recebendo *feedbacks*, perspectivas externas e comentários valiosos. Por sua vez, houve um processo de reflexão crítica permanente, complementado pelo estabelecimento de um ponto de saturação de dados, pela utilização de diários de campo e pelo registo de reflexões e emoções durante o processo, bem como pela adoção de uma abordagem transparente em todas as etapas da investigação.

### Análise de dados

Nas etapas iniciais, os pesquisadores analisaram individualmente as transcrições das entrevistas^22^ com a finalidade de obter o significado geral da experiência. Em seguida, foi realizada uma segunda leitura para identificação das unidades de significado. O “método comparativo constante”^23^ foi aplicado para chegar a um consenso na codificação das categorias^24^. A seguir, foi implementado um processo qualitativo de análise de dados^25^ em cinco fases: Acumulação, Desmontagem, Remontagem, Interpretação e Conclusão.

Durante a fase de Acumulação foi organizada e verificada a integridade das 18 entrevistas transcritas. Na fase de Desmontagem, os dados foram decompostos em elementos menores, o que envolveu uma leitura hermenêutica iterativa entre as partes e o todo. Isso facilitou a identificação de palavras, frases e narrativas recorrentes, possibilitando uma análise aprofundada na fase de Remontagem. Durante o processo de remontagem emergiram códigos que surgiram a partir do exercício interpretativo. No processo de Interpretação, o material remontado foi utilizado para construir uma “nova história”, extraindo diversos temas das entrevistas.

Os pesquisadores realizaram a codificação dos dados e identificaram conjuntamente as unidades de significado. A partir das relações entre essas unidades de significado, elas foram agrupadas em categorias abrangentes mais amplas, o que levou à criação de um sistema de núcleos temáticos ou metacategorias emergentes. Além disso, foram identificados vetores qualitativos para a interpretação dos dados por meio de uma análise sequencial e transversal das metacategorias com base no referencial teórico anterior, o que forneceu suporte empírico para a análise das informações. As discrepâncias surgidas durante a discussão foram abordadas e resolvidas^25^. Os dados foram gerenciados com o programa Atlas.ti 8.0. O plano de análise foi triangulado com os pesquisadores. Cinco participantes foram convidados a revisar as categorias identificadas que refletiam sua experiência como processo de confirmabilidade de categorias e códigos.

### Confiabilidade

A confiabilidade dos dados foi estabelecida com base nos critérios de Lincoln e Guba^26^, que incluem credibilidade, transferibilidade, confiabilidade e confirmabilidade. Os pesquisadores buscaram credibilidade interagindo continuamente com os participantes durante a coleta de dados e verificando as transcrições e códigos extraídos com os entrevistados para garantir sua precisão. A confirmabilidade foi assegurada através da análise independente dos dados por dois dos investigadores, que corroboraram os resultados sem se basearem em hipóteses próprias na interpretação dos dados e sem excluir quaisquer resultados que contrariassem as opiniões dos participantes. Em termos de confiabilidade, todas as entrevistas e a coleta de dados foram realizadas pelos mesmos pesquisadores, enquanto a avaliação e confirmação dos resultados foram revisadas por um pesquisador externo familiarizado com pesquisas qualitativas. Para alcançar a transferibilidade, utilizou-se uma amostragem com a máxima diversidade em termos de idade, ambos os sexos e níveis de escolaridade, além de fornecer um relato preciso das declarações dos participantes para garantir a aplicabilidade dos resultados em outros contextos.

### Aspectos éticos

A pesquisa aplicou os princípios éticos de acordo com a Declaração de Helsinque^27^, código CIOMS e requisitos éticos de Ezekiel Emanuel, sendo considerada como pesquisa de risco mínimo. Os participantes receberam e assinaram um Termo de Consentimento Livre e Esclarecido, no qual lhes foi explicado detalhadamente o objetivo do estudo, e foram sanadas quaisquer dúvidas que surgissem sobre os princípios da confidencialidade. Foram utilizados códigos para identificar cada participante, a fim de manter seu anonimato durante todas as etapas da pesquisa. O projeto de pesquisa foi aprovado pelo Comitê de Ética da Universidade do Cauca, conforme Ata nº 6.1-1.25/06 de março de 2023.

## Resultados

### Características dos participantes

Participaram da pesquisa 18 enfermeiros, cujas idades variaram entre 27 e 48 anos, com média de 38,8 anos. Em média, os entrevistados tinham 13 anos de experiência. As características sociodemográficas são apresentadas na Figura 1.

**Figura 1:** Caracterização sociodemográfica dos participantes. Percepção dos enfermeiros sobre o seu papel na redução das desigualdades em saúde em contextos comunitários. Colômbia, 2023

N.°	Educação	Idade	Gênero	Região	Anos de experiência
1	Mestre em Epidemiologia	36	Feminino	Cauca	13
2	Mestrado em Saúde Pública	38	Feminino	Nariño	16
3	Mestrado em Saúde Pública	41	Feminino	Cundinamarca	14
4	Mestre em Epidemiologia	38	Feminino	Cauca	16
5	Mestrado em Administração em Saúde	39	Feminino	Cauca	15
6	Mestrado em Saúde Pública	48	Masculino	Vale	21
7	Mestrado em Gestão	27	Feminino	Amazonas	3
8	Enfermeira Profissional	33	Feminino	Quibdô	5
9	Mestrado em Ambientes Hospitalares	44	Masculino	Huíla	10
10	Mestrado em Saúde Pública	48	Feminino	Risaralda	17
11	Mestrado em Ambientes Virtuais de Aprendizagem	36	Masculino	Caldas	20
12	Mestre em Epidemiologia	37	Feminino	Quindío	8
13	Especialista em Auditoria em Saúde	32	Feminino	Tolima	6
14	Enfermeira Profissional	35	Feminino	Tolima	7
15	Enfermeira Profissional	49	Feminino	Vale	23
16	Enfermeira Profissional	29	Feminino	Orinoquia	4
17	Especialista em Epidemiologia	46	Feminino	Atlántico	23
18	Especialista em Auditoria da Qualidade e Saúde	43	Feminino	Meta	14

Os participantes entrevistados perceberam que o papel que desempenharam em vários contextos comunitários durante a sua prática profissional permitiu-lhes contribuir significativamente para a redução das desigualdades em saúde das pessoas, famílias e comunidades.

Da análise dos dados emergiram um total de 379 códigos. Os pesquisadores interpretaram os códigos de forma independente e as categorias foram trianguladas. Foram identificados três temas principais (metacategorias): (a) Competências para construir, operacionalizar, monitorar e avaliar políticas de saúde; (b) Profissionais que geram estratégias para o cuidado da saúde e da vida; e (c) Abordagem de saúde e cuidado baseada nas características do território. Cada metacategoria possuía de duas a cinco categorias (Figura 2).

**Figura 2:** Metacategorias e categorias emergentes. Percepção dos enfermeiros sobre o seu papel na redução das desigualdades em saúde em contextos comunitários. Colômbia, 2023

As categorias que emergiram e as áreas de desigualdades em saúde nas quais os enfermeiros identificaram o seu papel na redução dessas desigualdades são detalhadas a seguir.

### Metacategoria: Competências para construir, operacionalizar, monitorar e avaliar políticas de saúde

No contexto da política de saúde, os entrevistados percebem que os enfermeiros ocupam cargos que lhes permitem tomar decisões na construção, implementação e avaliação de planos, programas e projetos relacionados à saúde, seguindo a estrutura do sistema de saúde colombiano. A participação dos enfermeiros nesta área estabelece critérios para definir ações que serão incluídas nos mandatos dos planos de desenvolvimento, tanto em nível municipal como departamental. Isto tem um impacto significativo nas orientações que serão seguidas para alocar recursos de saúde.

Cinco categorias emergem desta metacategoria e são descritas abaixo.

### Categoria: Definem ações de saúde pública em planos de ação nos níveis municipal, departamental, de seguros e hospitalar

Equipes lideradas por enfermeiros desenvolvem as ações que são incorporadas aos planos, programas e projetos a serem executados durante o período de governo (quatro anos), tanto no nível da secretaria municipal de saúde quanto no nível departamental. Da mesma forma, nas seguradoras e nos hospitais, esses profissionais contribuem para a definição dos processos estratégicos que servirão de base para a realização das iniciativas missionárias estabelecidas pelos diversos atores do sistema de saúde em seus esforços políticos. Nessa perspectiva, emergem discursos como os seguintes:

*Neste sistema de saúde, o enfermeiro tem a possibilidade de construir as políticas que serão implementadas nas secretarias e municípios. O mesmo acontece com os hospitais de primeiro nível: aí estamos pensando em como se consolidam os processos administrativos e assistenciais para avançar na garantia do direito à saúde* [...] *vejo que nas EPS (seguradoras) tem também muitos enfermeiros que dia após dia trabalham para garantir medicamentos, consultas e exames médicos aos beneficiários* [...] *é como uma rede de enfermeiros para alcançar o bem-estar das pessoas de todas as instituições de saúde* [...] *é assim Sinto que se contribui realmente para reduzir essas desigualdades na saúde.* (ETV1015M-48)

### Categoria: Executam planos, programas e projetos de atores de saúde

Os participantes percebem a sua liderança não só na fase de planejamento das ações, mas também na execução dos processos operacionais, onde desempenham um papel crucial na definição das diversas formas de execução das ações dos planos de desenvolvimento sanitário por parte dos atores. A implementação destes componentes da política pública é sistemática e liderada pelos enfermeiros, que, pelo seu papel, aproximam o sistema de saúde das populações que enfrentam maiores desafios no acesso aos planos de benefícios de saúde:

*Na Enfermagem se mobiliza um bom número de profissionais que se dedicam à execução de projetos de saúde pública na comunidade. Esse é um verdadeiro suporte que garante que as coisas sejam feitas com qualidade [...] porque se a gente olhar para outros profissionais, eles não têm esse suporte técnico, e talvez duvide se eles vão fazer as coisas conforme o planejado [...] quando a gente vê que tem enfermeiros no comando das instituições, sabe que haverá mais segurança, mais tranquilidade na obtenção de resultados para as pessoas.* (ETV1002F-27)

### Categoria: Auditam e monitoram políticas de saúde

Outro papel que o enfermeiro assume é a tarefa de avaliar o grau de cumprimento das ações implementadas pelos atores do sistema de saúde. Os participantes perceberam que a auditoria é um elemento crucial para alcançar os objetivos do sistema. Isto implica um processo de supervisão contínuo e sistemático para garantir o cumprimento da política de saúde, a fim de assegurar resultados satisfatórios para a população:

*As secretarias de saúde utilizam diferentes formatos de monitoramento e uma multiplicidade de indicadores com os quais monitoramos o que as entidades de saúde vêm executando* […] *essa é uma questão muito importante porque tomamos decisões com base nesses avanços e atuamos para melhorar a saúde das pessoas* [...] *e, é claro, a equidade na saúde a partir dessas evidências.* (ETV1005M-44)

### Categoria: Avaliam ações dos atores de saúde

Os participantes perceberam que a análise das informações e o progresso na implementação das ações de saúde permitem a avaliação das atividades realizadas pelos atores de saúde. Esta análise do grau de cumprimento das ações conduz à adaptação das atividades de acordo com as dinâmicas populacionais que caracterizam os territórios, com o objetivo de identificar oportunidades de melhoria na formulação dos planos, de forma a alcançar uma maior eficiência:

*Somos responsáveis por prestar assistência técnica às instituições de saúde dos municípios; Nesta tarefa vamos avaliar como estão progredindo na execução dos seus planos de desenvolvimento e os acompanhamos para que tanto os programas de saúde quanto as verbas atribuídas sejam executados de acordo com o que foi desenhado* [...] *depois fazemos um julgamento para verificar se o que foi projetado surtiu ou não efeito, tanto com os profissionais quanto com as comunidades* [...] *aquele cara a cara com as pessoas é muito emocionante e enche de satisfação se tudo deu certo* [...] *ou de raiva quando a gente vê que as coisas não foram feitas com dedicação.* (ETV1009M-36)

### Categoria: Lideram processos de saúde e cuidado em nível individual e coletivo

De acordo com as diretrizes da política de saúde, a gestão da qualidade nos atores de saúde é um requisito legal obrigatório. Os participantes percebem que são os enfermeiros que lideram de forma particular estes componentes da política, a partir da mobilização de recursos, estratégias e processos para garantir a segurança na prestação de serviços de saúde. Da mesma forma, percebem que garantir o cumprimento dos protocolos de cuidado e assistência às pessoas em ambientes comunitários após internação hospitalar ou em ações de promoção da saúde e prevenção de doenças é uma tarefa diária que contribui para a redução dos riscos para a saúde da população. A implementação dos planos de saúde individuais dos hospitais de baixa complexidade é realizada por enfermeiros, que reconhecem estruturas operacionais em nível comunitário para facilitar o acesso das pessoas aos serviços de saúde, com foco em ações de promoção da saúde e prevenção de doenças. Por outro lado, elaboram planos de saúde pública que envolvem intervenções coletivas e abordagens de risco nos níveis departamental e municipal. Por meio de diversas ações, implementam planos, programas e projetos que envolvem as comunidades em processos educativos que visam a prevenção de doenças ajustados às particularidades de cada contexto:

*Sempre tem um enfermeiro que está organizando tudo para que esses processos sejam cumpridos. ; Em geral, está sempre lá aguardando os programas para que eles continuem trabalhando, monitorando, medindo indicadores, dando feedback para as instituições, inclusive, muitas vezes temos que zelar pela questão dos custos e bom, nós temos que estar aí* [...] *e a gente pensa: isso tem suas vantagens, isso garante que as coisas funcionem.* (ETV1003F-41)

### Metacategoria: Profissionais que geram estratégias de cuidado à saúde e à vida

Os participantes percebem que os enfermeiros desenham estratégias para implementar ações de saúde e cuidado. Esses profissionais possuem visão integral do processo saúde-doença-atenção-cuidado; neste sentido, distinguem-se pela sólida ligação ontológica com as dinâmicas específicas do território, que utilizam para obter um maior impacto na redução das desigualdades em saúde.

### Categoria: Definem ferramentas de saúde comunitária 

Os participantes relatam a constante criação de ferramentas para colocar em prática a gestão da saúde comunitária. Isto envolve a integração do conhecimento local, redes comunitárias, participação social e colaboração com instituições de outros setores, como educação, infraestruturas e nutrição, entre outros:

*Como enfermeiro de saúde comunitária, é você quem deve criar a estratégia que melhor se adapta à população... claro, sempre considerando a voz das pessoas e os seus conhecimentos... o mesmo acontece quando se junta às demais instituições que administram os programas de educação, nutrição, esporte, água potável etc.* [...] *a gente fica pensando: que instrumento eu posso inventar para melhorar a saúde das pessoas?* […] *e o melhor de tudo é que as próprias pessoas iluminam a gente sobre o que fazer.* (ETV1012F-35)

### Categoria: Dinamizam processos educacionais

Um papel essencial do enfermeiro no âmbito comunitário é a sua contribuição para os processos educativos, onde se percebe que desempenham um papel predominante como agentes mobilizadores de informação e educação para a promoção da saúde e prevenção de doenças por meio do envolvimento de teorias e modelos pedagógicos para melhorar o ensino e a aprendizagem desta disciplina:

*Basicamente, da educação eu penso que é nisso que se concentra a nossa força* [...] *educar nos municípios e departamentos, educar a comunidade em geral, mas também poder orientar os profissionais que fazem parte tanto dos hospitais como dos municípios para que, por sua vez, eles possam abordar os problemas com a sua comunidade.* (ETV1014F-38)

### Categoria: Assumem o processo saúde-doença-atenção-cuidado de diversas formas

A percepção dos enfermeiros sobre o processo saúde-doença-atenção-cuidado traz implicações na forma como as ações são abordadas no ambiente comunitário. Isto não só é assumido na perspectiva da política de saúde, mas também incorpora as particularidades do território como elemento essencial para planear atividades ajustadas às dinâmicas socioculturais das populações:

*Sinto que felizmente nós enfermeiros consideramos a saúde como uma questão complexa e ampla, não só do ponto de vista clínico, mas também como resultado daquilo que é mobilizado em toda a comunidade* [...] *depende dos determinantes sociais* […] *essa perspectiva é muito importante para nós, como líderes do setor da saúde, avançarmos no fortalecimento das intervenções de saúde, especialmente da saúde comunitária* [...] *e referindo-nos ao que as pessoas manifestam e têm nas suas comunidades.* (ETV1018F-46)

### Metacategoria: Abordagem de saúde e cuidado a partir das características dos territórios

Segundo a percepção dos participantes, os enfermeiros levam em consideração a dinâmica sociocultural, política e econômica dos territórios para desenvolver ações de forma contextualizada, alinhadas aos parâmetros estabelecidos pelo sistema de saúde. Este trabalho é fundamental, pois não só implica um exercício ativo na implementação de planos de desenvolvimento institucional, mas também integra as realidades das populações sob diversas perspectivas. Isto inclui não apenas aspectos orçamentais e estratégicos, mas também recursos locais que contribuem para a gestão do processo de saúde.

### Categoria: Incorporam ações de cuidado diferenciadas

No processo de assistência à população, o enfermeiro estabelece critérios e desenha processos que facilitam a implementação de ações de cuidado diferenciadas. Estas ações são realizadas tanto na prestação de serviços individuais como no desenvolvimento de atividades coletivas nas comunidades:

*Devemos compreender essas outras formas do processo saúde-doença. Compreender, por exemplo, as desarmonias e desequilíbrios dos povos indígenas e afros e poder participar dessas outras construções do cuidado à saúde* [...] *ter alternativas ou formas alternativas de cuidar da saúde também contribui nesse processo de redução das desigualdades* [...]*. Diante desses contextos, estando em um ambiente tão rico culturalmente, o território se torna um sujeito que fala conosco.* (ETV1007F-36)

### Categoria: Fortalecem mecanismos de participação comunitária

Os enfermeiros reforçam o processo de participação comunitária ao estabelecer relações no território com diversas lideranças, organizações e atores sociais. Estas interações baseiam-se num diálogo colaborativo que permite pactuar ações consensuais com a comunidade. É neste contexto que o sistema de saúde, a população, os determinantes sociais e as lideranças comunitárias se integram para promover a mobilização de ações relacionadas com os cuidados de saúde:

*Quem mais senão nós para mobilizar a comunidade* [...] *somos nós que estamos mais próximos das pessoas, a quem concedem o valor da escuta ativa para coordenar e realizar atividades de saúde com as pessoas a partir dos seus sentimentos... nós organizamos com a comunidade as estratégias de acordo com as suas necessidades e bem, levamos adiante* [...] *sinto que isso nos mobiliza e nos faz sentir pessoas que contribuem para eliminar as desigualdades.* (ETV1011F-37)

Os participantes acrescentaram a percepção de que, apesar dos esforços realizados pelos atores que intervêm na saúde para abordar as desigualdades na saúde, persistem desafios substanciais que impedem o progresso sistemático nesse sentido. Estes desafios estão relacionados com o investimento limitado em programas de promoção da saúde, redução de salários, interrupções na contratação de profissionais, obstáculos no acesso aos serviços de saúde, burocracia excessiva na prestação de serviços, negação de cuidados de saúde, baixa disponibilidade de serviços em áreas rurais e práticas corruptas.

Por outro lado, embora a maioria dos participantes possuísse pós-graduação, esta característica não se revelou como requisito essencial para desempenhar o papel de enfermeiro que contribui para a redução das desigualdades em saúde. Na verdade, no seu processo de contratação, as instituições de saúde não costumam exigir requisitos específicos de formação em nível de pós-graduação.

## Discussão

A partir do exercício de compreender a percepção dos enfermeiros sobre o seu papel na redução das desigualdades em saúde nos ambientes comunitários, esses profissionais reconhecem a sua contribuição para esse objetivo a partir dos diferentes ambientes onde desenvolvem a sua prática profissional. Isto é conseguido por meio de diversas práticas de cuidado individual e coletivo, em ambiente domiciliar e comunitário, seguindo uma lógica voltada para a promoção da saúde e prevenção de doenças. As contribuições tornam-se evidentes na implementação de políticas de saúde definidas nos planos de desenvolvimento dos atores da saúde nos níveis municipal, departamental e nacional.

Apesar desta realidade, o reconhecimento pelo sistema de saúde do trabalho dos enfermeiros em contextos comunitários não está totalmente alinhado com a sua percepção. De fato, alguns sistemas de saúde restringem as intervenções dirigidas a estes contextos em termos estratégicos, financeiros, de recursos humanos e de infraestruturas, dando prioridade a processos de cuidados centrados em uma concepção hospitalocêntrica. Esta situação desencoraja a implementação sistemática da saúde comunitária, o que acarreta restrições no acesso ao sistema de saúde com as correspondentes implicações na redução das desigualdades^10^ ^),(^ ^12^. Da mesma forma, impacta negativamente no desempenho do enfermeiro ao assumir seu papel, pois afeta aspectos como sua remuneração, estabilidade no emprego, modalidades contratuais e consistência na implementação de estratégias de saúde comunitária^6^.

Por sua vez, outros estudos^28^ são consistentes com os resultados e destacam o papel dos enfermeiros na redução das desigualdades em saúde, abordando desafios críticos de saúde em nível macro que afetam persistentemente grupos de pessoas desfavorecidas a partir da ligação entre prevenção, tratamento e cuidados de saúde de forma integral, com ênfase na pessoa no contexto de sua vida e nas condições em que nasce, vive, trabalha e envelhece.

Para ilustrar a percepção dos enfermeiros sobre o seu papel na redução das desigualdades, três áreas-chave são incorporadas ao debate: a área da saúde, a área socioeconômica/cultural e o contexto^19^.

### Área do sistema de saúde

Além de avaliar as necessidades da população e participar do desenvolvimento de políticas de saúde, o enfermeiro desempenha um papel essencial na garantia do direito à saúde^16^ ^),(^ ^29^. Isto implica assumir responsabilidades administrativas e de gestão destinadas a promover o acesso, a utilização dos serviços, a continuidade, a oportunidade e a qualidade dos cuidados, princípios que permeiam qualquer sistema de saúde. Embora esta categoria surja com força nos relatos, diversas pesquisas indicam que muitas vezes os enfermeiros são excluídos da tomada de decisões na formulação de políticas públicas^30^ ^)-(^ ^31^, o que acarreta limitações na adoção de uma abordagem holística do processo saúde-doença nas ações de governança, com efeitos restritos na implementação de estratégias que promovam o bem-estar de pessoas em situações socioeconômicas desfavoráveis^6^. Apesar dessas discrepâncias, alguns estudos apoiam a ideia de que o enfermeiro desempenha um papel fundamental no exercício da política pública de saúde, ao participar de cenários que exigem tomadas de decisão de alcance mais amplo^10^.

Os enfermeiros distinguem-se pela capacidade de propor e desenvolver diversas estratégias de cuidados de saúde, tanto em nível individual, familiar e comunitário. Numa perspectiva ampla do processo saúde-doença-cuidado, possuem competências para analisar os determinantes sociais da saúde, o que lhes permite atuar por meio de práticas que visam a redução das desigualdades de acordo com os desafios particulares de cada território^13^. Porém, abordar os determinantes sociais implica assumir papéis e responsabilidades de outros setores e o protagonismo do enfermeiro pode ficar comprometido, principalmente se não houver um exercício político articulado que estabeleça os parâmetros pelos quais caminharão as ações do setor saúde em forma conjunta com outras instituições que contribuam para o bem-estar das populações^10^.

O monitoramento e a avaliação são elementos essenciais nos sistemas de saúde, destinados a avaliar o progresso das atividades na área da saúde. Com base nos dados recolhidos na dinâmica do setor da saúde, os enfermeiros analisam sistematicamente os indicadores e realizam avaliações críticas das intervenções quanto à sua eficácia. Ao identificar desvios no cumprimento dos objetivos, gerenciam essas restrições e, por meio de processos de melhoria, propõem estratégias que fortaleçam o cumprimento dos propósitos essenciais do sistema de saúde^32^. Uma das vantagens para alcançar esta função essencial da Enfermagem deve-se ao esforço destes profissionais em manter laços estreitos com as pessoas, as famílias e as comunidades, bem como com as instituições de saúde e a forma como o sistema de saúde é mobilizado a partir da lógica territorial^6^. Um aspecto complexo dentro desta dinâmica refere-se à capacidade das instituições em cumprir os objetivos estabelecidos nos planos de desenvolvimento. De alguma forma, estes objetivos estão condicionados pelas condições financeiras e estruturais de cada ator na área da saúde. Essa situação gera tensões nos processos de apoio dos enfermeiros durante a execução do monitoramento e avaliação^12^.

Um elemento fundamental da atuação do enfermeiro é a sua capacidade de liderar grupos de gestão em saúde, de promover ações individuais e coletivas. Essa habilidade é reforçada pela imersão sistemática que os profissionais de Enfermagem realizam nos territórios. Essa prática permite identificar as particularidades tanto do contexto quanto das instituições de saúde, o que por sua vez serve de base para promover ações a partir da saúde pública^33^ e das práticas avançadas de enfermagem^34^. Apesar da relevância dessas práticas, a liderança do grupo é afetada por desafios na coordenação das ações com profissionais de outras disciplinas devido às diferenças nas abordagens e perspectivas para assumir os processos de cuidado^35^. Por outro lado, muitas vezes há limitações na participação comunitária devido à falta de conscientização, desconfiança nos serviços de saúde e recursos limitados para participar ativamente, aspecto que pode desestabilizar o alcance do objetivo de saúde^6^. A isso, somamos as limitações no financiamento das equipes de saúde, a resistência a mudanças organizacionais e as altas cargas de trabalho que podem comprometer as ações de liderança do enfermeiro^12^.

É fundamental destacar o papel dos enfermeiros como agentes-chave na promoção de processos educativos em diversas frentes^6^. Na comunidade, desempenham um papel relevante na promoção de estilos de vida saudáveis e colaboram rigorosamente com os líderes comunitários para convocar e mobilizar as populações. Essa prática apoia a continuidade do processo educativo, quer seja a partir de ações individuais nos serviços de saúde ou com ações coletivas desenvolvidas em ambientes comunitários. Da mesma forma, prestam assessoria técnica a outros profissionais do sistema de saúde para impulsionar a política de saúde, conforme corroborado em outros estudos^7^. Este papel pode ser afetado por barreiras ambientais relacionadas ao tempo limitado para a realização de ações educativas, à descontinuidade dos planos e projetos de promoção da saúde, às competências pedagógicas limitadas dos profissionais de saúde e às restrições na comunicação devido a dialetos locais ou barreiras culturais que os profissionais de saúde abordam e incorporam de forma limitada^36^.

### Área socioeconômica/cultural

Os enfermeiros mobilizam ações que influenciam os ambientes sociais, dinamizando processos focados na saúde pública, a fim de reduzir os efeitos adversos das doenças e avançar na mitigação das desigualdades em saúde. Esse papel posiciona o enfermeiro como agente de mudança na organização da sociedade e como executor de metas de saúde baseadas em evidências científicas^35^ ^),(^ ^37^, em consonância com as necessidades da realidade social. Da mesma forma, os enfermeiros realizam análises minuciosas para identificar e selecionar os grupos populacionais mais desfavorecidos e, em seguida, concentram os seus esforços na melhoria do acesso ao sistema de saúde para esses grupos. Esta priorização estende-se a segmentos da população como mulheres, idosos, crianças, migrantes, grupos étnicos e pessoas com diversas orientações sexuais, entre outros. Este processo realça a relevância das contribuições da Enfermagem para a justiça social e a defesa dos direitos dos grupos vulneráveis^6^ ^),(^ ^13^ como resposta necessária às desigualdades em saúde e realça a responsabilidade social que recai sobre estes profissionais de saúde. Esta situação não seria problemática se os sistemas de saúde reconhecessem adequadamente o papel dos enfermeiros dentro desta complexa estrutura sociopolítica e conseguissem estabelecer elementos de políticas públicas que incentivassem a atuação destes profissionais. Esses elementos poderiam incluir melhorias salariais, atribuição de funções de liderança, oportunidades de formação de pós-graduação e modelos de saúde que fortaleçam os cuidados comunitários^6^.

Os enfermeiros conseguem adequar as ações de saúde e de cuidado às características específicas de cada território como parte integrante do seu trabalho na comunidade, considerando os capitais culturais, sociais, econômicos e simbólicos como recursos para promover iniciativas do setor da saúde. Assim, esses profissionais mergulham nas particularidades dos contextos em que atuam e adotam uma abordagem situada que reconhece o território como elemento dinâmico que influencia padrões de saúde, incapacidade e mortalidade^5^ ^),(^ ^8^. O desafio colocado por esta situação diz respeito às limitações no número de profissionais que optam por trabalhar nestes ambientes, especialmente em áreas rurais ou periurbanas. Nesses contextos, as atuais condições socioculturais e políticas oferecem poucas oportunidades para o desenvolvimento e fortalecimento da carreira profissional^7^.

O território é também reconhecido como uma componente essencial para o cuidado da saúde e da vida, susceptível de ser moldado para atingir um nível ótimo de saúde. Estas lógicas de cuidado no território por parte dos enfermeiros não se limitam a uma perspectiva estritamente técnica, mas incorporam uma sólida componente comunitária^7^ que fortalece a mobilização de processos e a liderança na comunidade. Ao reconhecerem esta dinâmica, os enfermeiros enfrentam a realidade de que nem todo o sistema de saúde funciona sob esta mesma lógica integrada. Por outro lado, as ações muitas vezes são realizadas de forma fragmentada, adaptando-se às contingências da dinâmica epidemiológica das populações atendidas. Esta situação implica um esforço adicional por parte dos profissionais de enfermagem, que devem enfrentar tanto os desafios inerentes ao trabalho comunitário como à coordenação das equipes de saúde sob uma premissa de ação coletiva^6^ ^),(^ ^12^.

### Área do contexto

Os enfermeiros identificam as desvantagens enfrentadas pelas pessoas, famílias e comunidades e tomam decisões a fim de facilitar o cuidado, tendo em conta as trajetórias de vida dos grupos populacionais. Neste processo, dão ênfase aos grupos com maior vulnerabilidade social e econômica, tendo em conta fatores como o contexto sócio-histórico, as políticas aplicadas, o nível educacional e as dinâmicas de violência social e política que caracterizam os territórios. Ao reconhecerem essas categorias, tomam decisões para implementar ações de saúde com especial ênfase no cuidado de populações com determinantes sociais complexos que limitam o acesso aos serviços de saúde. Estas ações são priorizadas em territórios com dificuldades de acesso geográfico, áreas afetadas por conflitos sociopolíticos, estratos socioeconômicos desfavorecidos e limitações na disponibilidade de serviços públicos. Embora estudos apoiem estes resultados^10^ ao indicar que na maioria dos países do mundo os enfermeiros são agentes de mudança que prestam apoio holístico àqueles cujos direitos e necessidades são negligenciados devido à sua situação social e são deixados numa condição de invisibilidade perante a sociedade, esta situação cria desafios para os profissionais continuarem a sua prática nestes cenários complexos onde necessitarão de elementos adicionais para apoiar não só a garantia de acesso aos cuidados de saúde, mas também ações contínuas para incentivar os enfermeiros a continuarem de acordo com esta dinâmica que desafia a permanência. no território e, devido a divergências no sistema de saúde, coloca em risco o cumprimento do papel do enfermeiro em cenários complexos^7^ ^),(^ ^12^ ^),(^ ^38^.

A contribuição deste estudo amplia a base de evidências científicas relacionadas à capacidade dos enfermeiros de influenciar a redução das desigualdades em saúde, bem como ao seu papel fundamental em ambientes onde a saúde e os cuidados comunitários são mobilizados. Estes cenários realçam a resiliência do enfermeiro, apesar de as políticas de saúde do país favorecerem uma abordagem de bem-estar. O estudo fornece bases para a construção e operacionalização de políticas que visem fortalecer as ações de enfermagem e enfatizar suas competências e liderança para enfrentar as desigualdades em saúde e melhorar o acesso, a equidade e a qualidade dos cuidados de saúde, especialmente aos grupos socioeconómicos e culturalmente desfavorecidos.

Dentro das limitações deste estudo, é importante destacar que a pesquisa se limita a um grupo de enfermeiros na Colômbia e que, consequentemente, as percepções e experiências descritas estão restritas ao contexto particular que foi objeto de análise. Nesse sentido, ressalta-se a necessidade de realizar estudos em outros contextos e ampliar a amostra, a fim de fortalecer a base de evidências.

## Conclusão

Os enfermeiros percebem que, por meio da sua prática profissional cotidiana em diferentes atores do sistema de saúde, contribuem significativamente para a redução das desigualdades em saúde em ambientes comunitários. Essas contribuições baseiam-se na concepção, desenho, operacionalização, monitoramento e avaliação de ações vinculadas aos planos, programas e projetos estabelecidos na política de saúde. Estes elementos da política são liderados tanto no nível regional como local por enfermeiros que, apoiados na sua formação na gestão de processos e construção de estratégias, conferem dinamismo ao sistema de saúde.

A mobilização de ações em âmbito comunitário pelos enfermeiros é determinada pelas características socioculturais, políticas e econômicas das populações. Estas variáveis são utilizadas como critérios para priorizar grupos vulneráveis, com o objetivo de melhorar o seu acesso aos serviços de saúde e reduzir as desigualdades.

# Translation: Percepción de enfermeros sobre su rol en la disminución de desigualdades en salud en contextos comunitarios

**DOI:** 10.1590/1518-8345.7245.4298

**Authors:** Jorge Sotelo-Daza, Yaneth Esperanza Jaramillo, Martha Vivas Chacón

**Affiliations:** 1 Universidad del Valle, Facultad de Salud, Cali, Colombia.; 2 Universidad del Área Andina, Facultad de Salud, Armenia, Colombia.; 3 Universidad del Cauca, Facultad Ciencias de la Salud, Popayán, Colombia.

**Keywords:** Enfermería, Disparidades en el Estado de Salud, Enfermería en Salud Pública, Determinantes Sociales de la Salud, Política de Salud, Investigación cualitativa

## Abstract

**Objetivo:**

comprender la percepción de los enfermeros sobre su rol en la disminución de desigualdades en salud en contextos comunitarios.

**Método:**

estudio cualitativo con enfoque fenomenológico abordado desde la teoría interpretativista de Heidegger y los ámbitos de las desigualdades en salud. Se realizaron 18 entrevistas a profundidad con enfermeros que se desempeñan en instituciones sanitarias de intervención comunitaria. Se adoptó el proceso de cinco fases para el análisis de datos cualitativos: Acumulación, Desmontaje, Reensamblaje, Interpretación y Conclusión.

**Resultados:**

se identificaron cuatro temas principales que movilizan los enfermeros para mitigar desigualdades en salud en escenarios comunitarios: Competencias para construir, operativizar, monitorear y evaluar la política de salud; Liderazgo y gestión de procesos de salud y del cuidado a nivel individual y colectivo; Profesionales generadores de estrategias para el cuidado y Enfoque de cuidado basado en características del territorio.

**Conclusión:**

los enfermeros perciben que su rol aporta de manera significativa a disminuir desigualdades en salud en escenarios comunitarios desde el diseño, la operación, el monitoreo y la evaluación de políticas de salud. A partir de las características socioculturales, económicas y políticas del territorio, priorizan acciones en grupos humanos en desventaja, a fin de mejorar el acceso, la oportunidad, la continuidad y la calidad en salud.

## Destacados

**(1)** Los enfermeros aportan de manera significativa a disminuir desigualdades en salud.

**(2)** La enfermería está arraigada en principios de equidad en salud y justicia social.

**(3)** La salud comunitaria denota efectividad en la reducción de desigualdades en salud.

**(4)** Las comunidades son esenciales en la lucha contra determinantes sociales adversos.

**(5)** Los enfermeros movilizan el diseño, la operación y la evaluación de políticas sanitarias.

## Introducción

Las desigualdades en salud son diferencias potenciales evitables en términos de salud o de riesgos para la salud, en los que las políticas sanitarias tienen la capacidad de influir entre grupos de personas que pueden estar más o menos favorecidas; estas diferencias colocan de manera sistemática a los grupos sociales desfavorecidos en mayor desventaja en materia de salud^1^. Las desigualdades en salud son el resultado de los Determinantes Sociales de la Salud (DSS), es decir, de las “circunstancias en que las personas nacen, crecen, trabajan, viven y envejecen, incluido el conjunto más amplio de fuerzas y sistemas que influyen sobre las condiciones de la vida cotidiana”^2^. Estas desigualdades surgen de la estratificación social basada en la riqueza, el poder y el prestigio, cuyos desbalances producen resultados de salud injustos en comunidades de bajo nivel socioeconómico a lo largo del tiempo^3^. Este concepto involucra dimensiones económicas, políticas, socioculturales y ambientales, por lo tanto, se ha descrito que solo es posible eliminar las desigualdades en salud a través de la acción sobre los DSS^4^. La lucha contra las desigualdades en salud alude a un problema de salud pública desafiante, pues determina las condiciones de bienestar de las poblaciones tanto en países pobres como en los ricos^5^.

En esta noción, a lo largo de la historia la Enfermería se ha erigido como una profesión arraigada en los principios de equidad en salud y justicia social cuyo papel preponderante ha sido mejorar la salud de las personas, familias y comunidades al abordar desigualdades sociales y de salud subyacentes^6^ ^)-(^ ^7^; sin embargo, el reconocimiento de esta labor por parte de actores de los sistemas sanitarios y de la sociedad ha estado relegado y se ve afectado por discursos y prácticas de lógicas asistencialistas que atenúan los relevantes aportes de los enfermeros en la dinamización de estrategias por fuera de escenarios hospitalarios.

La focalización de intervenciones a partir de la salud comunitaria se ha revelado como una estrategia efectiva para abordar desigualdades en salud^8^. Las comunidades locales desempeñan un papel esencial en la lucha contra los DDS adversos, en este sentido, se considera crucial empoderarlas y dotarlas de conocimientos y lograr compromisos para abordar los desafíos de salud y enfermedad de manera efectiva. El cuidado comunitario se presenta como un pilar fundamental de Enfermería^9^. A través de las relaciones que establecen con miembros de la comunidad, los enfermeros pueden influir en las conductas de salud de manera positiva y fomentar la adopción de comportamientos protectores para la salud^6^. De la misma manera, pueden empoderar a las comunidades al trabajar en la intersección de las políticas de salud de sus miembros y brindar soluciones efectivas y sostenibles^10^.

El personal de Enfermería en la comunidad opera desde perspectivas sistémicas e integra elementos conceptuales y prácticos para proporcionar atenciones a nivel individual y colectivo^11^. En calidad de profesión esencial en la promoción y educación para la salud, la Enfermería desempeña un papel crucial como agente estratégico para potenciar las condiciones de salud y minimizar los factores de riesgo. Este logro se materializa al promover comportamientos saludables en la vida cotidiana, a fin de aportar a la disminución de las desigualdades en salud^10^ ^),(^ ^12^. Fundamentada en los principios de derechos humanos y equidad, la ética de Enfermería respalda de manera sólida estas funciones prioritarias^6^.

El propósito de esta investigación yace en desentrañar la importancia del rol del enfermero en la reducción de las desigualdades en salud^13^, con respaldo en evidencia científica. Esto se fundamenta en diversas categorías, tales como garantizar el acceso al sistema de salud donde los enfermeros actúan como el primer contacto^10^, promover la educación y la salud^14^, brindar atención centrada en las personas^15^, participar en trabajo comunitario^16^ y coordinar el proceso de atención de manera integral^11^. En este contexto, el objetivo de la presente investigación fue comprender la percepción de los enfermeros sobre su rol en la disminución de desigualdades en salud en contextos comunitarios.

## Método

### Tipo de estudio

Estudio cualitativo con diseño descriptivo y exploratorio, donde se usó el enfoque fenomenológico interpretativista de Heidegger^17^, que prioriza las experiencias vividas y las percepciones fenomenológicas^18^ de los enfermeros en relación con la ejecución de su rol en escenarios comunitarios desde una perspectiva de desigualdad en salud^19^.

### Lugar y participantes del estudio

En el proceso de selección de los participantes se empleó un muestreo teórico propositivo o intencional^20^, que incluyó a 18 enfermeros que se desempeñaban como profesionales en contextos comunitarios procedentes de 14 regiones de Colombia. La cantidad de participantes se definió en función de alcanzar el punto en el que la recopilación de datos no proporcionó nuevos conceptos, categorías o relaciones. Los investigadores llevaron a cabo un proceso analítico reflexivo y crítico para evaluar los datos, con el fin de establecer el nivel de saturación teórica y la densidad de la información. Estos criterios se emplearon para vincular nuevos participantes tras un minucioso análisis sistemático de los datos recopilados. El período de estudio se extendió entre marzo y octubre de 2023.

Los criterios de inclusión considerados fueron los siguientes: trabajar en un contexto comunitario y contar con al menos dos años de experiencia en servicios del sistema de salud colombiano como hospitales, secretarías de salud municipales o departamentales, Ministerio de Salud y aseguradoras de salud.

Se exploraron las influencias de los enfermeros en la mitigación de las desigualdades en salud para profundizar en el significado de sus experiencias, tal como las viven en su desempeño diario. El estudio de la desigualdad en salud se abordó desde una perspectiva cualitativa^19^ y se consideraron tres ámbitos para el análisis: el ámbito de la salud, el socioeconómico/cultural y el contexto. Los investigadores emplearon la lista de verificación de Criterios Consolidados para Informar Investigaciones Cualitativas (*Consolidated Criteria for Reporting Qualitative research*, COREQ)^21^.

### Recolección de datos

Los datos se recopilaron a través de entrevistas en profundidad, llevadas a cabo tanto en reuniones presenciales en los sitios de trabajo de los participantes como en línea. Todas las entrevistas se grabaron en audio y, en el caso de las entrevistas en línea, en formato de video a través de la plataforma *Teams* ^*®*^ tras obtener el consentimiento de los participantes. Se realizaron transcripciones literales de las grabaciones utilizando el programa *Word* ^*®*^ , lo que permitió realizar los análisis primarios correspondientes. Antes de llevar a cabo la recopilación de datos, el autor principal realizó entrevistas piloto con el fin de garantizar claridad, coherencia y fluidez en la redacción y secuencia de las preguntas.

Las entrevistas no estuvieron sujetas a restricciones de tiempo y se desarrollaron de manera orgánica, guiadas por el compromiso de los participantes al relatar sus vivencias. Su duración osciló entre 57 y 93 minutos. El guión de la entrevista se elaboró sobre la base de la bibliografía relevante. Se tomaron notas de campo tanto durante como después de realizadas las entrevistas. En el momento de la investigación no había otras personas presentes además de los investigadores y los participantes. No se realizaron entrevistas repetidas. La recopilación de datos se dio por concluida cuando se alcanzó suficiencia y saturación muestral.

Durante las entrevistas, se pidió a los participantes que proporcionaran información como edad, nivel educativo, ocupación, cargo y tiempo de experiencia, seguido de preguntas abiertas que se formularon según una guía de entrevista. Todos los participantes respondieron a una pregunta inicial: “¿Puede decirme cómo percibe el rol del enfermero en la disminución de desigualdades en salud en contextos comunitarios?”. Se utilizaron sondeos y paráfrasis durante la recopilación de datos para garantizar que los participantes proporcionaran una visión adecuada de las preguntas realizadas. Otras preguntas planteadas fueron las siguientes: “¿Desde su percepción, qué aspectos del sistema de salud le han facilitado mitigar las desigualdades en salud?”, “¿Qué tipo de resistencias ha encontrado en el sistema de salud para mitigar las desigualdades en salud?”; “¿Podría describir su experiencia como enfermero/a en relación con la atención de personas en situación de vulnerabilidad social?”; y “¿Podría describir cómo ha abordado estas situaciones de desigualdad social desde su trabajo?”. Se avanzó en la conversación con comentarios como “Continúe”, “Por favor, cuénteme más sobre cómo fue para usted”, “¿Qué quiere decir con esta declaración?”, “¿Puede pensar en algo más que me ayude a entender cómo fue?” o “¿Le gustaría agregar algo más?”.

Uno de los componentes que cautelaron el rigor metodológico de la investigación estuvo relacionado con el equipo de investigadores compuesto por enfermeros de la Universidad del Valle, la Fundación Universitaria del Área Andina y la Universidad del Cauca. El equipo de investigadores cuenta con formación a nivel de Maestría en Salud Pública y Epidemiología y uno de ellos es Doctor en Antropología. También ejercen como docentes en estas instituciones y cuentan con amplia experiencia tanto en gestión de la salud pública en servicios del sistema de salud, así como en la realización de investigaciones a partir de métodos cualitativos. Dos de las investigadoras eran mujeres, mientras que uno era hombre. Los participantes estaban al tanto de los perfiles de los investigadores y reconocían su interés en explorar y comprender los aportes de los enfermeros en diferentes contextos a través de proyectos de investigación.

Las motivaciones personales que impulsaron la realización de esta investigación surgieron durante un simposio de Enfermería, en el cual los investigadores se congregaron para discutir asuntos pertinentes a los roles de los enfermeros en el ámbito de la salud pública. Preocupados por la limitada visibilización de los enfermeros en ciertas instituciones sanitarias, los investigadores coincidieron en que plantear esta investigación sería un aporte relevante en función de sus objetivos profesionales y experiencias previas en el ejercicio de la Enfermería.

Para que los participantes formaran parte de la investigación, el primer autor inició un contacto preliminar a través de correo electrónico (30 enfermeros), en el cual se detallaron los objetivos del estudio. Aquellas personas que expresaron interés en participar (21 enfermeros) fueron contactadas por teléfono para proporcionar información adicional sobre la investigación y coordinar los detalles de la entrevista, teniendo en cuenta su disponibilidad de tiempo, así como para acordar la fecha y el lugar de encuentro. Al final, tres personas decidieron no participar en la investigación debido a limitaciones de tiempo.

Los participantes de este estudio se desempeñaban en servicios del sistema de salud colombiano responsables de gestionar acciones a nivel comunitario. Estas entidades incluyen Secretarías de Salud Departamentales (regional) y Secretarías de Salud Municipales (local), además de instituciones de carácter público encargadas de liderar procesos de gestión de la salud pública de acuerdo con la política sanitaria nacional a través de planes, programas y proyectos. Otros participantes ejercían roles de liderazgo en hospitales públicos de baja complejidad (nivel 1) donde se implementan acciones de promoción de la salud y prevención de enfermedades en entornos rurales y urbanos. Además, algunos participantes trabajaban en aseguradoras de salud y en entidades privadas responsables de gestionar el riesgo y liderar el aseguramiento en salud de la población.

En el marco de la reflexividad, los investigadores reconocieron de manera crítica su papel durante todo el proceso de investigación y fueron conscientes de sus propias experiencias, valores y perspectivas para limitar su influencia en la recopilación e interpretación de los datos. Se adoptaron estrategias para minimizar sesgos a partir de la colaboración con otros investigadores, lo que permitió recibir retroalimentación, perspectivas externas y comentarios valiosos. A su vez, se vinculó un proceso de reflexión crítica continua, complementado con el establecimiento de un punto de saturación de datos, el uso de diarios de campo y el registro de reflexiones y emociones durante el proceso, así como la adopción de un enfoque transparente en todas las etapas de la investigación.

### Análisis de datos

En sus etapas iniciales, los investigadores realizaron el análisis de las transcripciones de las entrevistas de manera individual^22^ con el propósito de obtener el sentido general de la experiencia. Posterior a ello se llevó a cabo una segunda lectura para identificar las unidades de significado. Se aplicó el “método comparativo constante”^23^ para llegar a consensos en la codificación de categorías^24^. A continuación, se implementó un proceso de análisis de datos cualitativos^25^ en cinco fases: Acumulación, Desmontaje, Reensamblaje, Interpretación y Conclusión.

Durante la fase de Acumulación se organizó y verificó la integridad de las 18 entrevistas transcritas. En la fase de Desmontaje, los datos se desglosaron en elementos más pequeños, lo que implicó una lectura hermenéutica iterativa de ida y vuelta entre las partes y el todo. Esto facilitó la identificación de palabras, frases y narrativas recurrentes, lo que posibilitó el análisis en profundidad en la fase de Reensamblaje. Durante el proceso de reensamblaje emergieron códigos que surgieron del ejercicio interpretativo. En el proceso de Interpretación se utilizó el material reensamblado para construir una “nueva historia” extrayendo diversos temas de las entrevistas.

Los investigadores llevaron a cabo la codificación de los datos y, de manera conjunta, identificaron unidades de significado. A partir de relaciones entre estas unidades de significado, se agruparon en categorías comprensivas más amplias, lo que condujo a la creación de un sistema de núcleos temáticos emergentes o metacategorías. Además, se identificaron vectores cualitativos para la interpretación de los datos mediante un análisis secuencial y transversal de las metacategorías basado en el marco teórico previo, que dio el sustento empírico para el análisis de la información. Se abordó y resolvió cualquier discrepancia que surgió durante la discusión^25^. Los datos se gestionaron con el programa Atlas.ti 8.0. El plan de análisis se trianguló con los investigadores. Se invitó a cinco participantes a revisar las categorías identificadas que reflejaban su experiencia a modo de un proceso de confirmabilidad de categorías y códigos.

### Confiabilidad

La confiabilidad de los datos se estableció con base en los criterios de Lincoln y Guba^26^, que incluyen credibilidad, transferibilidad, confiabilidad y confirmabilidad. Los investigadores procuraron credibilidad a través de la interacción de manera continua con los participantes durante la recopilación de datos y a partir de la verificación de las transcripciones y códigos extraídos con los entrevistados para garantizar su precisión. La confirmabilidad se aseguró mediante un análisis independiente de los datos realizado por dos de los investigadores, quienes corroboraron los hallazgos sin basarse en sus propias hipótesis en la interpretación de los datos y sin excluir ningún resultado que contradijera la opinión de los participantes. En cuanto a la confiabilidad, todas las entrevistas y los datos recopilados estuvieron a cargo de los mismos investigadores, en tanto que la evaluación y confirmación de los hallazgos fueron revisados por un investigador externo familiarizado con la investigación cualitativa. Para lograr transferibilidad se utilizó un muestreo con la máxima diversidad en términos de edad, ambos géneros y niveles educativos, además de proporcionar un relato preciso de las declaraciones de los participantes para asegurar la aplicabilidad de los hallazgos en otros contextos.

### Aspectos éticos

La investigación aplicó los principios éticos conforme a la Declaración de Helsinki^27^, código CIOMS y requisitos éticos de Ezekiel Emanuel, considerándola como una investigación de riesgo mínimo. Los participantes recibieron y firmaron un consentimiento informado, en el cual se les explicó de forma detallada en relación con el propósito del estudio, y se resolvieron las dudas que surgieron acerca de los principios de confidencialidad. Se utilizaron códigos para identificar a cada participante con el fin de mantener su anonimato durante todas las etapas de la investigación. El proyecto de investigación fue aprobado por el Comité de Ética de la Universidad del Cauca, según Acta N.º 6.1-1.25/06 de marzo de 2023.

## Resultados

### Características de los participantes

En la investigación participaron 18 enfermeros cuyas edades oscilaron entre los 27 y 48 años, con una media de 38,8 años. En promedio, los entrevistados tenían 13 años de experiencia. Las características sociodemográficas se presentan en la Figura 1.

**Figura 1:** Caracterización sociodemográfica de los participantes. Percepción de enfermeros sobre su rol en la disminución de desigualdades en salud en contextos comunitarios. Colombia, 2023

N.º	Nivel educativo	Edad	Género	Región	Años de experiencia
1	Magíster en Epidemiología	36	Femenino	Cauca	13
2	Magister en Salud Pública	38	Femenino	Nariño	16
3	Magister en Salud Pública	41	Femenino	Cundinamarca	14
4	Magíster en Epidemiología	38	Femenino	Cauca	16
5	Magister en Administración en Salud	39	Femenino	Cauca	15
6	Magister en Salud Pública	48	Masculino	Valle	21
7	Magister en Gerencia	27	Femenino	Amazonas	3
8	Enfermera Profesional	33	Femenino	Quibdó	5
9	Magíster en Ambientes Hospitalarios	44	Masculino	Huila	10
10	Magister en Salud Pública	48	Femenino	Risaralda	17
11	Magíster en Ambientes Virtuales de Aprendizaje	36	Masculino	Caldas	20
12	Magíster en Epidemiología	37	Femenino	Quindío	8
13	Especialista en Auditoria en Salud	32	Femenino	Tolima	6
14	Enfermera Profesional	35	Femenino	Tolima	7
15	Enfermera Profesional	49	Femenino	Valle	23
16	Enfermera Profesional	29	Femenino	Orinoquía	4
17	Especialista en Epidemiología	46	Femenino	Atlántico	23
18	Especialista en Calidad y Auditoria en Salud	43	Femenino	Meta	14

Los participantes entrevistados percibieron que el papel que han desempeñado en varios entornos comunitarios durante su ejercicio profesional les ha permitido contribuir de manera significativa a la reducción de las desigualdades en salud de las personas, familias y comunidades.

Del análisis de datos surgieron un total de 379 códigos. Los investigadores interpretaron los códigos de forma independiente y se triangularon las categorías. Se identificaron tres temas principales (metacategorías): (a) Competencias para construir, operativizar, monitorear y evaluar la política de salud; (b) Profesionales generadores de estrategias para el cuidado de la salud y la vida; y (c) Enfoque de salud y cuidado basado en las características de los territorios. Cada metacategoría tenía de dos a cinco categorías (Figura 2).

**Figura 2:** Metacategorías y categorías emergentes. Percepción de los enfermeros sobre su rol en la disminución de desigualdades en salud en contextos comunitarios. Colombia, 2023

A continuación se detallan las categorías que emergieron y los ámbitos de las desigualdades en salud en los cuales los enfermeros identificaron su papel en la reducción de estas desigualdades.

### Metacategoría: Competencias para construir, operativizar, monitorear y evaluar la política de salud

En el contexto de la política de salud, los entrevistados perciben que los enfermeros ocupan posiciones que les permiten tomar decisiones en la construcción, implementación y evaluación de planes, programas y proyectos relacionados con el cuidado de la salud, siguiendo la estructura de la política del sistema sanitario colombiano. La participación de los enfermeros en este ámbito establece criterios para definir acciones que se incluirán en los mandatos de los planes de desarrollo, tanto a nivel municipal como departamental. Esto tiene un impacto significativo en las direcciones que se seguirán para asignar recursos en salud.

De esta metacategoría se desprenden cinco categorías que se describen a continuación.

### Categoría: Definen acciones en salud pública en planes de acción a nivel municipal, departamental, aseguradoras y hospitales

Equipos dirigidos por enfermeros desarrollan las acciones que se incorporan en planes, programas y proyectos a ejecutar durante el período gubernamental (cuatro años), tanto a nivel de secretarías de salud municipales como a nivel departamental. Asimismo, en las aseguradoras y los hospitales, estos profesionales contribuyen a definir los procesos estratégicos que servirán como base para llevar a cabo iniciativas misionales establecidas por los diversos actores del sistema de salud en sus esfuerzos políticos. En este orden de ideas surgen discursos como los siguientes:

*En este sistema de salud, los enfermeros tenemos la posibilidad de construir las políticas que se van a implementar en los departamentos y municipios. Lo mismo ocurre para los hospitales de primer nivel: allí estamos pensando cómo se consolidan los procesos administrativos y asistenciales para avanzar en la garantía del derecho a la salud* […] *veo que desde las EPS (aseguradoras) también hay muchos enfermeros que día a día trabajan para garantizarles los medicamentos, las consultas y los exámenes médicos a los afiliados* […] *es como una red de enfermeros para alcanzar el bienestar de la gente desde todas las instituciones de salud* […] *es así como siento que uno realmente aporta a disminuir tales desigualdades de la salud.* (ETV1015M-48)

### Categoría: Ejecutan planes, programas y proyectos desde los actores de salud

Los participantes perciben su liderazgo no solo en la fase de planificación de las acciones sino también en la ejecución de los procesos operativos, donde desempeñan un papel crucial al definir diversas formas de llevar a cabo las acciones de los planes de desarrollo por parte de los actores sanitarios. La implementación de estos componentes de la política pública es sistemática y está liderada por enfermeros, quienes, a través de su rol, acercan el sistema de salud a poblaciones que enfrentan mayores desafíos en el acceso a los planes de beneficios en salud:

*En la Enfermería se moviliza una buena cantidad de profesionales que se dedican a ejecutar los proyectos de salud pública en la comunidad; eso sí que es un verdadero soporte que garantiza que las cosas se hagan con calidad* […] *pues si uno mira a otros profesionales no tienen ese sustento técnico, y uno talvez duda si van a hacer las cosas según se han proyectado* […] *cuando uno ve que hay enfermeros al frente de las instituciones, va a tener más seguridad, más tranquilidad de lograr los resultados para la gente.* (ETV1002F-27)

### Categoría: Auditan y monitorizan la política de salud

Desde otro rol, los enfermeros asumen la tarea de evaluar el grado de cumplimiento de las acciones implementadas por actores del sistema de salud. Los participantes percibieron que la auditoría es un elemento crucial para alcanzar los objetivos del sistema. Esto implica un proceso continuo y sistemático de supervisión para garantizar el cumplimiento de la política sanitaria, a fin de asegurar resultados satisfactorios para la población:

*Desde las secretarías de salud se utilizan diferentes formatos de seguimiento y una batería de indicadores con los que hacemos el monitoreo de lo que las entidades de salud han ido ejecutando* […] *esto es un tema muy importante porque tomamos decisiones según esos avances y actuamos para lograr mejorar la salud de la gente* […] *y claro, la equidad en salud a partir de esas evidencias.* (ETV1005M-44)

### Categoría: Evalúan acciones de los actores en salud

Los participantes percibieron que el análisis de la información y el progreso en la implementación de acciones de salud les permiten llevar a cabo una evaluación de las actividades realizadas por los actores sanitarios. Este análisis del grado de cumplimiento de las acciones conduce a adaptar las actividades según las dinámicas poblacionales que caracterizan los territorios, con el objetivo de identificar oportunidades de mejora en la formulación de planes, a fin de alcanzar una mayor eficiencia:

*Nosotros nos encargamos de realizar la asistencia técnica a las instituciones de salud de los municipios; en esa tarea vamos y evaluamos cómo van avanzando en la ejecución de sus planes de desarrollo y acompañamos para que tanto los programas de salud y los presupuestos asignados se ejecuten según lo que se ha diseñado* […] *luego hacemos un juicio para verificar si lo que se proyectó surtió efecto o no, tanto con los profesionales como con las comunidades* […] *ese cara a cara con la gente es muy emocionante y lo llena a uno de satisfacción si todo ha ido bien* […] *o de rabia cuando uno ve que las cosas no se han hecho con dedicación. (ETV1009M-36)*

### Categoría: Lideran procesos de salud y del cuidado a nivel individual y colectivo

De acuerdo con las directrices de la política sanitaria, la gestión de la calidad en los actores de salud es un requisito normativo de cumplimiento obligatorio. Los participantes perciben que son los enfermeros quienes lideran estos componentes de política de manera particular, a partir de la movilización de recursos, estrategias y procesos para garantizar la seguridad en la prestación de servicios de salud. Del mismo modo, perciben que garantizar el cumplimiento de protocolos de atención y asistencia a las personas en entornos comunitarios posterior a una estancia hospitalaria o en acciones de promoción de la salud y prevención de enfermedades es una tarea cotidiana que contribuye a reducir los riesgos para la población. La implementación de planes de salud individuales desde los hospitales de baja complejidad está a cargo de enfermeros, quienes reconocen estructuras operativas a nivel comunitario con el fin de facilitar el acceso de las personas a servicios de salud, enfocadas en acciones de promoción de la salud y prevención de enfermedades. Por otra parte, elaboran los planes de salud pública que involucran intervenciones colectivas y con enfoques de riesgo desde los niveles departamental y municipal. A través de diversas acciones, implementan planes, programas y proyectos que involucran a las comunidades en procesos educativos orientados a la prevención de enfermedades ajustadas a las particularidades de cada contexto:

*Siempre hay un enfermero que está organizando todo para que se cumplan esos procesos; por lo general siempre está ahí pendiente de los programas para que sigan funcionando, haciendo seguimiento, midiendo indicadores, retroalimentando a las instituciones, inclusive, hasta muchas veces nos toca velar por el tema de costos y bueno, nos corresponde estar como en todo* […] *y uno piensa: eso tiene sus ventajas, eso garantiza que las cosas funcionen.* (ETV1003F-41)

### Metacategoría: Profesionales generadores de estrategias para el cuidado de la salud y la vida

Los participantes perciben que los enfermeros diseñan las estrategias para implementar acciones de salud y cuidado. Estos profesionales poseen una visión integral del proceso de salud-enfermedad-atención-cuidado; en ese sentido, se distinguen por su sólida conexión ontológica con las dinámicas específicas del territorio, que emplean para lograr un mayor impacto en la reducción de las desigualdades en salud.

### Categoría: Definen herramientas de salud comunitaria

Los participantes relatan la constante creación de herramientas para poner en práctica la gestión comunitaria en salud. Esto implica integrar conocimientos locales, redes comunitarias, participación social y colaboración con instituciones de otros sectores, como educación, infraestructura y nutrición, entre otros:

*A uno como enfermero desde la salud comunitaria le compete cranearse la estrategia que mejor cale en la población… eso sí, siempre del lado de la voz de la gente y sus saberes... lo mismo pasa al unirse con las otras instituciones que manejan los programas de educación, nutrición, deportes, agua potable etcétera* […] *uno piensa y piensa: ¿qué instrumento me invento para mejorar la salud de la gente?* […] *y lo mejor es que la misma gente le da luces para hacerlos.* (ETV1012F-35)

### Categoría: Dinamizan procesos educativos

Un papel esencial de los enfermeros en el ámbito comunitario es su contribución a los procesos educativos, donde se percibe que desempeñan un papel preponderante como agentes que movilizan la información y la educación con el fin de promover la salud y prevenir enfermedades a través del involucramiento de teorías y modelos pedagógicos para mejorar la enseñanza y el aprendizaje desde esta disciplina:

*Básicamente desde la educación creo que en eso se centra nuestro fuerte* […] *educar en los municipios y los departamentos, educar a la comunidad en general pero también poder orientar a los profesionales que forman parte tanto de los hospitales como de los municipios para que, a su vez, ellos puedan abordar las temáticas con su comunidad.* (ETV1014F-38)

### Categoría: Asumen el proceso salud-enfermedad-atención-cuidado de diversas maneras

La percepción de los enfermeros acerca del proceso salud-enfermedad-atención-cuidado tiene implicaciones en la forma en que se abordan las acciones en el entorno comunitario. Esto no se asume solo desde la perspectiva de la política sanitaria, sino que también incorpora las particularidades del territorio como un elemento esencial para planificar actividades ajustadas a la dinámica sociocultural de las poblaciones:

*Siento que afortunadamente los enfermeros consideramos la salud como un tema complejo y amplio, no solo desde lo clínico, más vale lo vemos como un resultado de lo que se moviliza en toda la comunidad* […] *depende de los determinantes sociales* […] *esa mirada es muy importante para que, como líderes del sector salud, avancemos en el fortalecimiento de las intervenciones en salud, especialmente desde la salud comunitaria* […] *y haciendo referencia a lo que las personas manifiestan y tienen en sus comunidades. (ETV1018F-46)*

### Metacategoría: Enfoque de salud y cuidado basado en las características de los territorios

Según la percepción de los participantes, los enfermeros tienen en cuenta las dinámicas socioculturales, políticas y económicas de los territorios para desarrollar acciones de manera contextualizada, alineadas con los parámetros establecidos por el sistema de salud. Esta labor es fundamental, ya que no solo implica un ejercicio activista en la implementación de los planes institucionales de desarrollo, sino que también integra las realidades de las poblaciones desde diversas perspectivas. Esto incluye no solo los aspectos presupuestarios y estratégicos, sino también recursos locales que contribuyen a gestionar el proceso de salud.

### Categoría: Incorporan acciones de atención diferencial

En el proceso de proporcionar asistencia a la población, el enfermero establece criterios y diseña procesos que facilitan la implementación de acciones de atención diferenciadas. Estas acciones se llevan a cabo tanto en la prestación de servicios individuales como en el desarrollo de actividades colectivas en comunidades:

*Hay que comprender esas otras formas del proceso de salud-enfermedad. De entender, por ejemplo, las desarmonías y los desequilibrios indígenas y afros y poder participar en esas otras construcciones de cuidado de la salud* […] *tener alternativas o formas alternas de cuidar la salud también nos aporta en ese proceso de disminuir desigualdades* […] *al enfrentarme a estos contextos, el estar en un ambiente tan rico culturalmente, el territorio se vuelve un sujeto que me habla.* (ETV1007F-36)

### Categoría: Fortalecen mecanismos de participación comunitaria

Los enfermeros refuerzan el proceso de participación comunitaria al establecer relaciones en el territorio con diversos líderes, organizaciones y actores sociales. Estas interacciones se basan en un diálogo colaborativo que permite acordar las acciones consensuadas con la comunidad. Es en estos contextos que se integran el sistema de salud, la población, los determinantes sociales y el liderazgo comunitario para promover la movilización de acciones relacionadas con la atención en salud:

*Quién más que nosotros para movilizar a la comunidad* […] *somos los que estamos más cerca de la gente, a quienes nos inculcaron el valor de la escucha activa para concertar y concretar las actividades de salud con la gente desde su sentir… organizamos con la comunidad las estrategias según sus necesidades y bueno, las sacamos adelante* […] *siento que esto nos mueve y nos hace sentir personas que aportamos para eliminar desigualdades.* (ETV1011F-37)

Los participantes añadieron la percepción de que, a pesar de los esfuerzos realizados desde los actores sanitarios para abordar las desigualdades en salud, persisten desafíos sustanciales que obstaculizan el avance sistemático hacia este fin. Estos desafíos se relacionan con una limitada inversión en programas de promoción de la salud, salarios reducidos, interrupciones en la contratación de profesionales, obstáculos en el acceso a los servicios de salud, excesiva burocracia en la prestación de servicios, negación de atenciones sanitarias, baja disponibilidad de servicios en áreas rurales y prácticas corruptas.

De otra parte, aunque la mayoría de los participantes poseían estudios de posgrado, dicha característica no se reveló como un requisito indispensable para desempeñarse en el rol de enfermeros que aportan a la reducción de las desigualdades en materia de salud. De hecho, en su proceso de contratación, las instituciones sanitarias no suelen exigir requisitos específicos de formación a nivel de posgrado.

## Discusión

Desde el ejercicio de comprender la percepción de los enfermeros sobre su rol en la disminución de las desigualdades en salud en entornos comunitarios, estos profesionales reconocen su contribución a este propósito desde los diferentes escenarios donde realizan su ejercicio profesional. Esto se logra a través de diversas prácticas en la atención individual y colectiva, en los entornos domiciliario y comunitario, siguiendo una lógica centrada en la promoción de la salud y la prevención de enfermedades. Los aportes se hacen evidentes en la implementación de políticas de salud que se definen en los planes de desarrollo de los actores sanitarios a nivel municipal, departamental y nacional.

A pesar de esta realidad, el reconocimiento de la labor de los enfermeros en contextos comunitarios por parte del sistema sanitario no se encuentra plenamente alineada con su percepción. De hecho, algunos sistemas de salud restringen las intervenciones dirigidas a estos contextos en términos estratégicos, financieros, de recursos humanos e infraestructura, priorizando en su lugar procesos asistencialistas de carácter hospitalocéntrico. Esta situación desincentiva la implementación sistemática de la salud comunitaria, lo cual conlleva restricciones en el acceso al sistema de salud con las correspondientes implicaciones en la reducción de desigualdades^10^ ^),(^ ^12^. Del mismo modo, repercute de manera negativa en el desempeño del enfermero al asumir su rol, ya que afecta aspectos como su remuneración, estabilidad laboral, modalidades contractuales y consistencia en la implementación de estrategias de salud comunitarias^6^.

Por su parte, otros estudios^28^ son consistentes con los hallazgos y destacan el papel de los enfermeros en la reducción de las desigualdades en salud al abordar los desafíos críticos de salud a un nivel macro que afectan persistentemente a grupos de personas en desventaja a partir de la vinculación de procesos de prevención, tratamiento y atención de manera integral, con énfasis en la persona en el contexto de su vida y las condiciones en las que nace, vive, trabaja y envejece.

Para elucidar la percepción de los enfermeros sobre su rol en la disminución de desigualdades, se incorporan tres ámbitos clave al debate: el ámbito de la salud, el socioeconómico/cultural y el contexto^19^.

### Ámbito del sistema de salud

Además de evaluar las necesidades de la población y participar del desarrollo de políticas sanitarias, los enfermeros desempeñan un papel esencial en la garantía del derecho a la salud^16^ ^),(^ ^29^. Esto implica asumir responsabilidades administrativas y de gestión orientadas a promover el acceso, uso de servicios, continuidad, oportunidad y calidad de la atención, principios que permean cualquier sistema de salud. Aunque esta categoría emerge con fuerza en los relatos, diversas investigaciones indican que los enfermeros a menudo son excluidos de la toma de decisiones en la formulación de políticas públicas^30^ ^)-(^ ^31^, lo que conlleva limitaciones en la adopción de un enfoque holístico hacia el proceso de salud-enfermedad en las acciones de gobernanza, con efectos restringidos en la implementación de estrategias que promuevan el bienestar de las personas en situaciones socioeconómicas desfavorables^6^. A pesar de estas discrepancias, ciertos estudios respaldan la idea de que los enfermeros desempeñan un papel fundamental en el ejercicio de la política pública sanitaria al participar en escenarios que demandan una toma de decisiones de alcance más amplio^10^.

Los enfermeros se distinguen por su habilidad para proponer y desarrollar diversas estrategias de atención sanitaria, tanto a nivel individual y familiar como comunitario. Desde una perspectiva amplia del proceso de salud-enfermedad-atención, tienen competencias para analizar determinantes sociales de la salud, lo que les permite actuar a través de prácticas destinadas a reducir desigualdades de acuerdo con los retos particulares de cada territorio^13^. Ahora bien, el abordaje de los determinantes sociales implica asumir roles y responsabilidades por parte de otros sectores y el papel protagónico del enfermero puede verse comprometido, sobre todo si no se dispone de un ejercicio político articulado que establezca los parámetros por donde van a transitar las acciones del sector salud en conjunto con otras instituciones que aportan al bienestar de las poblaciones^10^.

El monitoreo y la evaluación son elementos esenciales en los sistemas de salud, destinados a evaluar el progreso de las actividades sanitarias. Basados en datos recopilados de la dinámica del sector salud, los enfermeros analizan sistemáticamente indicadores y realizan evaluaciones críticas de las intervenciones en términos de su efectividad. Al identificar desviaciones en el logro de los objetivos, gestionan estas restricciones y, mediante procesos de mejora, plantean estrategias que fortalezcan el cumplimiento de los propósitos esenciales del sistema sanitario^32^. Una de las ventajas para lograr esta función esencial de la Enfermería obedece al esfuerzo de estos profesionales por mantener vínculos estrechos tanto con las personas, familias y comunidades, así como con las instituciones sanitarias y la manera como se moviliza el sistema sanitario desde la lógica territorial^6^. Un aspecto complejo dentro de esta dinámica hace referencia a la capacidad de las instituciones para cumplir con los objetivos establecidos en los planes de desarrollo. De alguna manera, estos objetivos están condicionados por las condiciones financieras y estructurales de cada actor en el ámbito de la salud. Esta situación genera tensiones en los procesos de acompañamiento de los enfermeros durante la ejecución del monitoreo y la evaluación^12^.

Un elemento fundamental del rol del enfermero es su capacidad para liderar grupos de gestión en salud, para promover acciones tanto individuales como colectivas. Esta habilidad se ve reforzada por la inmersión sistemática que los profesionales de Enfermería llevan a cabo en los territorios. Esta práctica les permite identificar las particularidades tanto del contexto como de las instituciones de salud, lo que a su vez les sirve como base para promover acciones desde la salud pública^33^ y la práctica avanzada en enfermería^34^. A pesar de la relevancia de estas prácticas, el liderazgo de los grupos se ve afectado por retos en la coordinación de las acciones con profesionales de otras disciplinas debido a las diferencias en enfoques y perspectivas para asumir los procesos de atención^35^. Por otra parte, a menudo existen limitaciones en la participación de la comunidad debido a falta de conciencia, desconfianza en los servicios de salud y recursos limitados para participar de manera activa, aspecto que puede desestabilizar el logro del propósito sanitario^6^. A esto se le suman limitaciones en el financiamiento de los equipos de salud, resistencias a los cambios organizacionales y altas cargas de trabajo que pueden comprometer las acciones de liderazgo del enfermero^12^.

Es esencial destacar el papel de los enfermeros como agentes clave en la promoción de procesos educativos desde diferentes frentes^6^. En la comunidad, desempeñan un papel relevante en el fomento de estilos de vida saludables y colaboran de manera rigurosa con líderes comunitarios para convocar y movilizar a las poblaciones. Esta práctica respalda la continuidad del proceso educativo, ya sea desde acciones individuales en servicios de salud o desde acciones colectivas desarrolladas en entornos comunitarios. Del mismo modo, proporcionan asesoramiento técnico a otros profesionales del sistema de salud con el fin de dinamizar la política sanitaria, tal como lo corroboran otros estudios^7^. Este rol puede verse afectado por barreras del entorno relacionadas con el limitado tiempo para realizar las acciones de educación, la discontinuidad de los planes y proyectos de promoción de la salud, las limitadas competencias pedagógicas de los profesionales de salud y las restricciones en la comunicación debido a dialectos locales o barreras culturales que el sistema de salud aborda e incorpora de manera limitada^36^.

### Ámbito socioeconómico/cultural

Los enfermeros movilizan acciones que influyen en los entornos sociales a partir de la dinamización de procesos centrados en la salud pública, con el fin de reducir los efectos adversos de las enfermedades y avanzar en la mitigación de las desigualdades en salud. Este rol posiciona al enfermero como un agente de cambio en la organización de la sociedad y como ejecutor de metas en salud basadas en la evidencia científica^35^ ^),(^ ^37^, en consonancia con las necesidades de la realidad social. Del mismo modo, los enfermeros realizan análisis minuciosos para identificar y seleccionar a los grupos de población más desfavorecidos, para luego enfocar sus esfuerzos en mejorar el acceso al sistema de salud para estos grupos. Esta priorización se extiende a segmentos de la población como mujeres, ancianos, niños, migrantes, grupos étnicos y personas con diversas orientaciones sexuales, entre otros. Este proceso subraya la relevancia del aporte de la Enfermería a la justicia social y la defensa de los derechos de grupos vulnerables^6^ ^),(^ ^13^ como una respuesta necesaria a las desigualdades en salud y resalta la responsabilidad social que recae sobre estos profesionales sanitarios. Esta situación no sería problemática si los sistemas de salud reconocieran adecuadamente el papel de los enfermeros dentro de esta estructura sociopolítica compleja, y lograran establecer elementos de política pública que incentiven el desempeño de estos profesionales. Estos elementos podrían incluir mejoras salariales, asignación de roles de liderazgo, oportunidades de formación de posgrado y modelos de salud que fortalezcan la atención comunitaria^6^.

Los enfermeros logran adaptar las acciones de salud y cuidado a las características específicas de cada territorio como parte integral de su labor en la comunidad considerando los capitales culturales, sociales, económicos y simbólicos como recursos para impulsar las iniciativas desde el sector salud. Así las cosas, estos profesionales se sumergen en las particularidades de los contextos en los que operan y adoptan un enfoque situado que reconoce al territorio como un elemento dinámico que influye en los patrones de salud, discapacidad y mortalidad^5^ ^),(^ ^8^. El desafío que plantea esta situación se refiere a las limitaciones en la cantidad de profesionales que eligen trabajar en estos entornos, especialmente en áreas rurales o periurbanas. En tales contextos, las condiciones socioculturales y políticas presentes ofrecen pocas oportunidades para el desarrollo y fortalecimiento de su carrera profesional^7^.

El territorio también se reconoce como un componente esencial para el cuidado de la salud y la vida, susceptible de ser moldeado para lograr un óptimo nivel de salud. Estas lógicas de atención en el territorio por parte de los enfermeros no se limitan a una perspectiva estrictamente técnica, sino que incorporan un componente comunitario sólido^7^ que fortalece la movilización de procesos y el liderazgo en la comunidad. Al reconocer estas dinámicas, los enfermeros se enfrentan a la realidad de que no todo el sistema sanitario opera bajo esta misma lógica integrada. En cambio, muchas veces las acciones se llevan a cabo de manera fragmentada, adaptándose a las contingencias de la dinámica epidemiológica de las poblaciones atendidas. Esta situación implica un esfuerzo adicional por parte de los profesionales de Enfermería, quienes deben afrontar tanto los desafíos inherentes al trabajo comunitario como la coordinación de los equipos de salud bajo una premisa de acción colectiva^6^ ^),(^ ^12^.

### Ámbito del contexto

Los enfermeros identifican las desventajas que enfrentan las personas, familias y comunidades y toman decisiones con el fin de facilitar la atención, teniendo en cuenta las trayectorias de vida de los grupos poblacionales. En este proceso, hacen hincapié en los grupos de mayor vulnerabilidad social y económica teniendo en cuenta factores como el contexto sociohistórico, las políticas aplicadas, el nivel educativo y las dinámicas de violencia social y política que caracterizan a los territorios. Al reconocer estas categorías, toman decisiones para implementar las acciones de salud con especial énfasis en la atención de poblaciones con determinantes sociales complejos que limitan el acceso a los servicios de salud. Estas acciones se priorizan en territorios con dificultades en el acceso geográfico, áreas afectadas por conflictos sociopolíticos, bajos estratos socioeconómicos y limitaciones en la disponibilidad de servicios públicos. Si bien estudios respaldan estos hallazgos^10^ al indicar que en la mayoría de los países del mundo los enfermeros son agentes de cambio que brindan apoyo holístico a aquellos cuyos derechos y necesidades son pasados por alto debido a su situación social y quedan en condición de invisibilidad ante la sociedad, esta situación genera desafíos para que los profesionales continúen su ejercicio en estos escenarios complejos donde van a requerir de elementos adicionales para apoyar no solo la garantía del acceso a la atención sanitaria, sino también acciones continuas para incentivar al enfermero a continuar de acuerdo con esta dinámica que reta la permanencia en el territorio y, por desavenencias del sistema sanitario, se pone en riesgo el cumplimiento del rol del enfermero en escenarios complejos^7^ ^),(^ ^12^ ^),(^ ^38^.

La contribución de este estudio amplía la base de evidencia científica relacionada con la capacidad de los enfermeros para influir en la reducción de las desigualdades en salud, así como su papel fundamental en escenarios donde se moviliza la salud y el cuidado comunitario. Estos escenarios destacan la resiliencia del enfermero, a pesar de que las políticas sanitarias del país favorezcan un enfoque asistencialista. El estudio proporciona un fundamento para la construcción y operacionalización de políticas orientadas a fortalecer el actuar enfermero y hacer hincapié en sus competencias y liderazgo para abordar las desigualdades en salud y mejorar el acceso, la equidad y la calidad de la atención en salud, especialmente a grupos en desventaja socioeconómica y cultural.

Dentro de las limitaciones de este estudio, es importante destacar que la investigación se circunscribe a un grupo de enfermeros en Colombia y que, en consecuencia, las percepciones y experiencias descritas están restringidas al contexto particular que fue objeto de análisis. En este sentido, se plantea la necesidad de llevar a cabo estudios en otros contextos y de ampliar la muestra, con el propósito de robustecer la base de evidencia.

## Conclusión

Los enfermeros perciben que, a través de su ejercicio profesional cotidiano en diferentes servicios del sistema sanitario, aportan de manera significativa a reducir las desigualdades en salud en escenarios comunitarios. Estas contribuciones se basan en la concepción, el diseño, la operacionalización, la monitorización y la evaluación de acciones vinculadas en los planes, programas y proyectos establecidos en la política de salud. Estos elementos de política son liderados tanto a nivel regional como local por los enfermeros, quienes, apoyados en su formación para la gestión de procesos y construcción de estrategias, dotan de dinamismo al sistema sanitario.

La movilización de las acciones en escenarios comunitarios por parte de los enfermeros está determinada por características socioculturales, políticas y económicas de las poblaciones. Estas variables se utilizan como criterios para priorizar a los grupos en situación de vulnerabilidad, con el objetivo de mejorar su acceso a los servicios de salud y reducir las desigualdades.
